# *Plasmodium* infection fully activates the immune system in peripheral blood and tumor microenvironment in a murine Lewis lung cancer model

**DOI:** 10.3389/fmolb.2025.1724792

**Published:** 2026-01-28

**Authors:** Qunfeng Huang, Dongheng Yang, Zhu Tao, Zhaoqing Yu, Wenting Ding, Na Tao, Xiulin Liao, Haoxin Lin, Zhipeng Cheng, Susu Gao, Zhongkui Kang, Jianming Xie, Wen Hu, Li Qin, Xiaoping Chen, Guifang Yu

**Affiliations:** 1 Guangdong Engineering Technology Research Center for Biological Targeting Diagnosis, Therapy, and Rehabilitation, Department of Oncology, The Fifth Affiliated Hospital, Guangzhou Medical University, Guangzhou, China; 2 CAS Lamvac (Guangzhou) Biomedical Technology Co., Ltd., Guangzhou, China; 3 School of Basic Medical Sciences, Guangzhou Medical University, Guangzhou, China; 4 State Key Laboratory of Respiratory Disease, Guangzhou Institutes of Biomedicine and Health, Chinese Academy of Science, Guangzhou, China

**Keywords:** high-dimensional flow cytometry, immune profiles, immunotherapy, non-small cell lung cancer, *Plasmodium* infection, tumor ecosystem

## Abstract

**Background:**

*Plasmodium* infection has been proven to activate antitumor immune responses. This study comprehensively analyzes the immune cell populations in peripheral blood and tumor microenvironment to elucidate the potential immunological mechanisms by which *Plasmodium* infection inhibits tumor growth.

**Methods:**

We established a subcutaneous Lewis lung cancer model in C57BL/6J mice and treated them with intraperitoneal injection of *Plasmodium yoelii*. The long and short diameters of tumors were measured. Then, high-dimensional flow cytometry was used to analyze the T cell subsets, macrophages and myeloid-derived suppressor cells (MDSCs) in peripheral blood and tumor tissues. Immunosuppression-related phosphorylated signal transducer and activator of transcription 3 (pSTAT3) and TGFβ in tumor tissues were also measured through Western blotting assay.

**Results:**

*Plasmodium* infection inhibited the growth of Lewis lung cancer in mice. The infection increased in the numbers of CD3^+^ T cells, including CD4^+^ and CD8^+^ T, CD4^+^ central memory T (Tcm), CD4^+^ effector memory T (Tem), CD8^+^ Tcm, CD8^+^ Tem, CD8^+^ virtual memory T (Tvm), CD8^+^ short-lived effector cells (SLEC), and CD8^+^ memory precursor effector cells (CD8^+^ MPEC) in peripheral blood. Concurrently, the infection also increased the numbers of CD3^+^ T cells, including CD4^+^ and CD8^+^ T, CD4^+^ Tcm, CD4^+^ Tem, CD4^+^ tissue resident memory T (Trm), CD8^+^ Tcm, CD8^+^ Tem, CD8^+^ Trm and CD8^+^ SLEC in tumor tissues. In addition, *Plasmodium* infection reduced the expression levels of PD-1 on CD4^+^ and CD8^+^ T, the number of polymorphonuclear MDSCs, and increased the ratio of M1/M2 macrophages in the tumor tissues. The initial mechanism study revealed that *Plasmodium* infection significantly reduced the expression levels of pSTAT3 and TGFβ in tumor tissues, providing direct evidence that *Plasmodium* infection activated the antitumor immune responses.

**Conclusion:**

Based on our past and current studies, we can draw the following conclusion: *Plasmodium* infection fully remodels and activates the immune system, targets and inhibits the entire tumor ecosystem through the key signals of pSTAT3 and TGFβ. This is completely different from the mechanisms of action of the current immune checkpoint blockade therapies, representing a new form of cancer immunotherapy, namely, the immune ecotherapy.

## 1 Introduction

Lung cancer remains the leading cause of cancer-related deaths worldwide (Bray et al., 2024). Non-small cell lung cancer (NSCLC) accounts for approximately 85% of all lung cancer cases, presenting significant therapeutic challenges (Bray et al., 2024). Despite recent advances in targeted therapies and immunotherapies, the 5-year overall survival rate for NSCLC remains low at about 22.9% (Schabath and Cote, 2019). While immunotherapy, particularly immune checkpoint inhibitors, has shown promise, its efficacy is limited to a subset of patients, highlighting the need for novel immunotherapeutic approaches (Wang et al., 2022).

The interaction between the tumor microenvironment and systemic immune responses plays a crucial role in cancer progression and therapy outcomes (Hiam-Galvez et al., 2021). Within the tumor microenvironment, T cell subsets, including CD4^+^ and CD8^+^ T cells, are key players in antitumor immunity (Ahmed et al., 2023). CD8^+^ T cells, particularly effector memory CD8^+^ T cells (Tem), are critical for direct tumor cell killing (Ganesan et al., 2017). Myeloid-derived suppressor cells (MDSCs) and tumor-associated macrophages (TAMs) often contribute to an immunosuppressive microenvironment, hindering effective antitumor responses (Kumar et al., 2016; Wang et al., 2019). Concurrently, the composition and function of immune cells in peripheral blood can reflect systemic immune status and potentially predict treatment responses (Spitzer et al., 2017). However, the complex interplay between these immune cell populations in both the tumor microenvironment and peripheral blood of NSCLC patients, and their potential modulation for therapeutic benefit, remains incompletely understood.

Recent studies have explored the potential of *Plasmodium* infection in cancer immunotherapy (Chen et al., 2021; Chen et al., 2011; Liu et al., 2017; Adah et al., 2019; Qin et al., 2020; Wang B. et al., 2020; Liang et al., 2021; Pan et al., 2021; Tao et al., 2022; Chen et al., 2023). Our previous research demonstrated that *Plasmodium* infection can inhibit tumor growth and metastasis in cancer models by activating innate and adaptive immune responses (Chen et al., 2011; Liang et al., 2021). We observed significant changes in T cell distribution and enhanced tumor-specific killing ability following *Plasmodium* infection (Chen et al., 2011; Pan et al., 2021; Tao et al., 2022). However, the specific effects of *Plasmodium* infection on T cell subsets in both peripheral blood and the tumor microenvironment, especially those representative T cell subsets such as naïve T cells, central memory T cells (Tcm), effector memory T cells (Tem), tissue-resident memory T cells (Trm), virtual memory T cells (Tvm), short-lived effector cells (SLEC), and memory precursor effector cells (MPEC), have neither been comprehensively characterized in our previous studies, nor addressed by any other investigators to date (Chen et al., 2021; Chen et al., 2011; Liu et al., 2017; Adah et al., 2019; Qin et al., 2020; Wang B. et al., 2020; Liang et al., 2021; Pan et al., 2021; Tao et al., 2022; Chen et al., 2023; Cossarizza et al., 2021; Muroyama and Wherry, 2021).

In this study, we aimed to investigate the effects of *Plasmodium* infection on distribution of T cell subsets, MDSCs and macrophage cells in peripheral blood and tumor microenvironment using high-dimensional flow cytometry in a mouse Lewis lung cancer model. At the same time, we measured the expression levels of PD-1 on these T cell subpopulations, and some key transcription factors and cytokines (such as pSTAT3 and TGFβ) perhaps related to these changes, in order to preliminarily explore the molecular mechanisms underlying these immune phenotype alterations. We hypothesized that *Plasmodium* infection would significantly alter composition and polarization of these cell populations towards more antitumoral phenotypes, potentially enhancing antitumor immune responses. This research provides new immunological evidence for the tumor-inhibiting effects of *Plasmodium* infection and may offer insights into novel immunotherapeutic strategies for lung cancer treatment.

## 2 Materials and methods

### 2.1 Mice

Six to eight-week-old C57BL/6J wild-type female mice were purchased from the Vital River Experiment Animal Company (Beijing, China). All mice were housed in a specific-pathogen-free (SPF) barrier facility at the Animal Center of Guangzhou Medical University. Animals were maintained under controlled conditions (12-h light/dark cycle, 22 °C ± 2 °C, 50% ± 10% relative humidity) with *ad libitum* access to food and water. All animal experiments were conducted in accordance with the Experimental Animal Ethics Guidelines of Guangzhou Medical University (approval number: GY 2022-076, approved on 28 April 2022).

### 2.2 Cell culture and parasites

The murine Lewis lung Cancer (LLC) cell line was obtained from the American Type Culture Collection (ATCC, Manassas, VA, USA). LLC cells were maintained in Roswell Park Memorial Institute 1,640 medium (RPMI-1640, Cat#SH30809.01B, HyClone, Logan, UT, USA) supplemented with 10% fetal bovine serum (FBS) and 1% penicillin-streptomycin (100 U/mL penicillin and 100 μg/mL streptomycin). Cells were cultured in a humidified incubator at 37 °C with 5% CO_2_ and passaged every 2–3 days or when reaching 80%–90% confluence.

The wild type murine *Plasmodium yoelii* 17XNL (Py) strain and *Plasmodium chabaudi* AS (Pc) were obtained from the Malaria Research and Reference Reagent Resource Center (MR4, BEI Resources, Manassas, VA, United States). The strain of attenuated Py was obtained through repeated passages of the wild type in mice. All Py and Pc were propagated by intraperitoneal injection into C57BL/6J mice. Parasitemia was monitored by examining Giemsa-stained (Cat#48900, Sigma-Aldrich, St. Louis, MO, USA) thin blood smears. Parasite density (%) was calculated by counting the number of *Plasmodium*-infected red blood cells (RBCs) in at least 1,000 RBCs.

### 2.3 Tumor model and animal grouping

C57BL/6J mice were subcutaneously inoculated with 5 × 10^5^ LLC cells in 100 μL phosphate-buffered saline (PBS) into the right flank. Mice were then randomly divided into two groups: LLC group (LLC) and LLC + Py group (n = 10 per group). One day after tumor inoculation, each mouse in the LLC + Py group was injected intraperitoneally with 5 × 10^5^ attenuated Py-infected RBCs, while each mouse in the LLC group was injected intraperitoneally with the same number of non-infected RBCs. Tumor dimensions were measured twice every week using digital calipers. Tumor volume was calculated using formula V = (ab^2^)/2, where V is the tumor volume, a is the longest diameter, and b is the shortest diameter. Animals were euthanized according to humane endpoints, including neurological dysfunction, hunched posture, lethargy, >20% weight loss, impaired mobility, or tumor size exceeding 1,500 mm^3^. The specific procedure of euthanasia involved the use of carbon dioxide (CO_2_) asphyxiation. Mice were exposed to rising levels of CO_2_ in a non-pre-filled cage. The CO_2_ was introduced at a displacement rate of 30%–70% of the cage volume per minute, adjusted according to the anesthesia process and the condition of the mice.

### 2.4 Preparation of single-cell suspensions

Peripheral blood samples: On day 16 post-tumor inoculation, mice were anesthetized through induce at 4% and maintenance at 2% isoflurane (Cat# R510-22-10, RWD, China) in a 30% oxygen and 70% nitrous oxide mixture using a small animal anesthesia machine (TAIJI-IE, RWD, China). And then approximately 200 μL of whole blood was collected via retro-orbital bleeding into tubes containing 200 μL of 3.8% sodium citrate. Samples were gently inverted to mix and prevent coagulation. RBCs were lysed using ammonium-chloride-potassium (ACK) lysis buffer (150 mM NH_4_Cl, 10 mM KHCO_3_, 0.1 mM EDTA, pH 7.2-7.4). The remaining cells were washed with PBS and collected by centrifugation (300 × g, 5 min, 4 °C) for flow cytometry staining.

Tumor tissue samples: On day 16 post-tumor inoculation, tumor tissues were excised, minced into small pieces (1–2 mm^3^), and placed into Hank’s balanced salt solution (HBSS) containing 1 mg/mL collagenase D (Cat#11088866001, Roche, Basel, Switzerland), 20 U/mL deoxyribonuclease I (DNase I, Cat#D5025, Sigma-Aldrich), and 3 mM CaCl_2_. Samples were digested for 30–60 min at 37 °C with gentle agitation. RBCs were lysed using ACK lysis buffer. The resulting cell suspension was filtered through a 70-μm nylon cell strainer (Cat#352350, Corning Falcon™, Corning, NY, USA), washed with 10 mL RPMI 1640 containing 2% FBS, and collected by centrifugation (300 × g, 5 min, 4 °C). Cells were resuspended in 100 μL PBS for flow cytometry staining.

### 2.5 Flow cytometry staining and analysis

Single-cell suspensions (1 × 10^6^ cells/sample) were incubated with Zombie Yellow™ Fixable Viability Kit (Cat#423106, BioLegend, San Diego, CA, United States) at 1:500 dilution in 100 μL PBS for 10 min at room temperature in the dark to assess cell viability. Cells were then washed with 2 mL flow cytometry staining buffer (Cat#554656, BD Biosciences, San Jose, CA, USA). To block Fc receptors, each sample was incubated with 0.25 μg TruStain FcX™ (anti-mouse CD16/32 antibody, Cat#156604, BioLegend), as shown in Supplementary Table S1 on ice in the dark for 10 min. After washing, cells were stained with fluorochrome-conjugated antibodies listed in the Supplementary Table S1 against surface markers for 30 min at 4 °C in the dark. The collected cells were added with 50 μL Precision Count Beads™ (Cat# 424902, Biolegend) per tumor or blood example to calculate the absolute number of cells. Prior to analysis, cells were filtered through a 200-mesh filter.

Flow cytometry was performed using a Cytek Aurora spectral flow cytometer (Cytek Biosciences, Fremont, CA, United States). Data were analyzed using FlowJo software v10.6 (Tree Star Inc., Ashland, OR, United States). The gating strategies for immune cell subpopulations for peripheral blood and tumor are respectively illustrated in Supplementary Figures S1, S2. Cell subpopulation abbreviations and corresponding surface markers are listed in Supplementary Table S2.

### 2.6 Western blotting for pSTAT3 and TGFβ measurement

On day 19 after tumor inoculation, tumor tissues were obtained and weighed, then homogenized and lysed using RIPA lysis buffer (Cat# KGP702-100, KeyGene), supplemented with a mixture of protease inhibitor cocktail (Cat# P1010, Beyotime) and phosphatase inhibitor cocktail D (Cat# P1096, Beyotime). This process was conducted on ice for 30 min, followed by centrifugation for collecting the supernatant samples. Protein concentration was determined by the bicinchoninic acid (BCA) assay (Cat# P0012, Beyotime). 60 μg protein samples were then subjected to polyacrylamide gel electrophoresis (Cat# M00665, GenScript), and subsequently transferred onto polyvinylidene fluoride membranes (Cat# ISEQ00010, Millipore). After treatment with the appropriate secondary antibodies, the protein bands were revealed using an enhanced chemiluminescence (ECL) detection kit (Cat# WBULS0500, Millipore) and visualized with a chemiluminescence imaging system (Tanon 5,200, Tanon Science and Technology, Shanghai, China). Band signal intensities were semi-quantitatively analyzed and densitometrically measured employing ImageJ 1.38 (NIH, https://imagej.nih.gov/ij/). Data were expressed as the ratio of densitometric values for proteins of interest and GAPDH protein. The antibodies utilized in this study included: anti-STAT3 (phosphor Y705) antibody (Cat#ab76315, Abcam); anti-TGF-β1 antibody (Cat#ab25121, Abcam), anti-GAPDH antibody (Cat#ab8245, Abcam); HRP-linked anti-mouse IgG H&L antibody (Cat#7076, CST); HRP-linked goat anti-rabbit IgG H&L antibody (cat#ab97051, Abcam).

### 2.7 Statistical analysis

Data are presented as mean ± standard error of the mean (SEM). Statistical significance between two independent groups was determined using unpaired two-tailed Student's t-tests or Mann-Whitney U test. All statistical analyses were performed using GraphPad Prism version 9.0 (GraphPad Software, San Diego, CA, USA). *P* values <0.05 were considered statistically significant. The levels of significance are denoted as follows: ns (not significant), * (*P* < 0.05), ** (*P* < 0.01), *** (*P* < 0.001), and **** (*P* < 0.0001).

## 3 Results

### 3.1 Impact of *Plasmodium* infection on tumor growth

**FIGURE 1:**
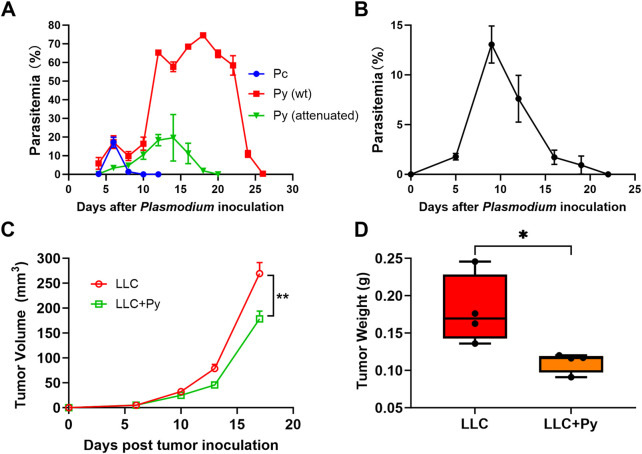
The dynamics of parasite density of different *Plasmodium* infections in mice, and the inhibitory effect of attenuated *Plasmodium yoelii* (attenuated Py) infection on tumor in a Lewis lung cancer model. **(A)** The parasitemia of wild type (wt) Py, attenuated Py, and *Plasmodium chabaudi* (Pc) in naïve mice (n = 3 or 4). **(B)** The parasitemia of attenuated Py in tumor-bearing mice (LLC + Py group, n = 10). **(C)** Tumor growth curves were recorded over a period of 17 days (n = 10 each group). **(D)** Tumor weight on day 16 post-tumor inoculation. Data are presented as mean ± SEM. Statistical significance was defined as *P* <0.05. Asterisks indicate statistically significant differences (*, *P* < 0.05; **, *P* < 0.01).

In our clinical studies of *Plasmodium* immunotherapy (*Plasmodium vivax* infection) for advanced cancers, we controlled the parasitemia at very low levels (Chen et al., 2021). Therefore, in this study, we conducted the experiment for the first time by using attenuated Py to simulate the clinical studies. The dynamic changes of parasitemia of wild-type and attenuated Py, as well as Pc in naïve mice are shown in Figure 1A. The dynamic change of parasitemia of attenuated Py in tumor-bearing mice is shown in Figure 1B. In the following text and Figures (the section of Results), “Py” specifically refers to the attenuated Py.

We firstly evaluated the effect of Py infection on tumor growth in a mouse Lewis lung cancer (LLC) model. C57BL/6J mice were divided into two groups: the tumor group (LLC group) and the treatment group (LLC + Py group). One day after the injection of tumor cells, the LLC + Py group were treated with Py-infected red blood cells, and the LLC group were treated with uninfected red blood cells as control. The results indicated that the tumor growth rate in the LLC + Py group is slower than that in the LLC group (*P* < 0.05, Figure 1C). In addition, on the 16th day after tumor inoculation, the tumor weight of the LLC + Py group was significantly lower than that of the LLC group (*P* < 0.05, Figure 1D). Py infection reduced the tumor burden by 27% based on tumor weight on the 16th day after tumor inoculation. The median survival time of tumor bearing mice in the LLC + Py group was significantly longer than that (27 days) in the LLC group (*P* = 0.02, Supplementary Figure S3A). The infection of attenuated Py had no significant effect on the body weight in the tumor-bearing mice (Supplementary Figure S3B). These results suggested that Py infection effectively inhibited tumor growth in Lewis lung cancer model, which is consistent with our previous research findings (Chen et al., 2011).

### 3.2 Changes in T cell subsets in peripheral blood

**FIGURE 2:**
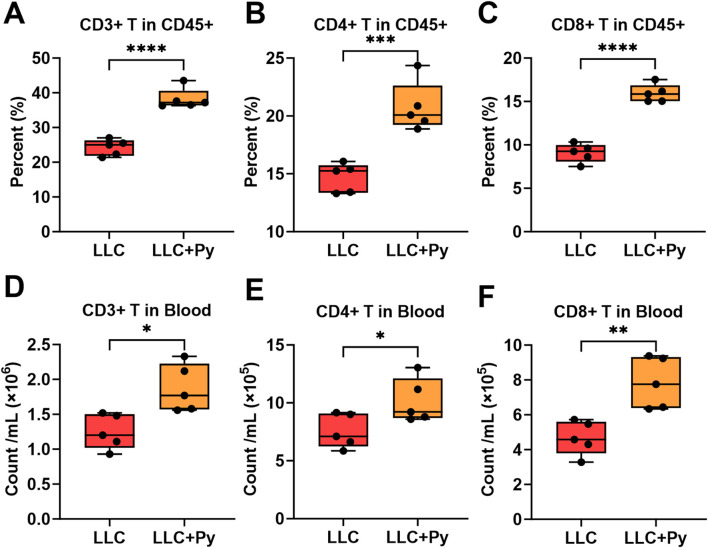
Impact of Py infection on T cells in peripheral blood. **(A)** Percentage of CD3^+^ T cells in the CD45^+^ cell population. **(B)** Percentage of CD4^+^ T cells in the CD45^+^ cell population. **(C)** Percentage of CD8^+^ T cells in the CD45^+^ cell population. **(D)** Count of CD3^+^ T cells per mL peripheral blood. **(E)** Count of CD4^+^ T cells per mL peripheral blood. **(F)** Count of CD8^+^ T cells per mL peripheral blood. Data are expressed as mean ± SEM (n = 5 per group). Asterisks indicate statistically significant differences (*, *P* < 0.05; **, *P* < 0.01, ***, *P* < 0.001; ****, *P* < 0.0001).

On day 16 post-tumor inoculation, high-dimensional flow cytometry was used to analyze T cell subsets in the peripheral blood. Firstly, Py infection significantly increased the percentage of CD3^+^ T cells in the CD45^+^ cells population (*P* < 0.05, Figure 2A). Secondly, Py infection significantly decreased the percentage of CD4^+^ T cells in the CD3^+^ T cell population (*P* < 0.05, Supplementary Figures S4, S5A), while significantly increased the percentage of CD8^+^ T cells in the CD3^+^ T cell population (Supplementary Figures S4, S5B). However, the infection significantly upregulated both the percentages of CD4^+^ and CD8^+^ T cells in the CD45^+^ cell population (both *P* < 0.05, Figures 2B,C). We then determined the absolute numbers of these immune cells in the peripheral blood, and found that there was no significant difference in the counts of CD45^+^ cells between the two groups (Supplementary Figure S5C), and that the counts of CD3^+^, CD4^+^ and CD8^+^ T cells in the LLC + Py group were significantly higher than those in the LLC group (all *P* < 0.05, Figures 2D–F).

**FIGURE 3:**
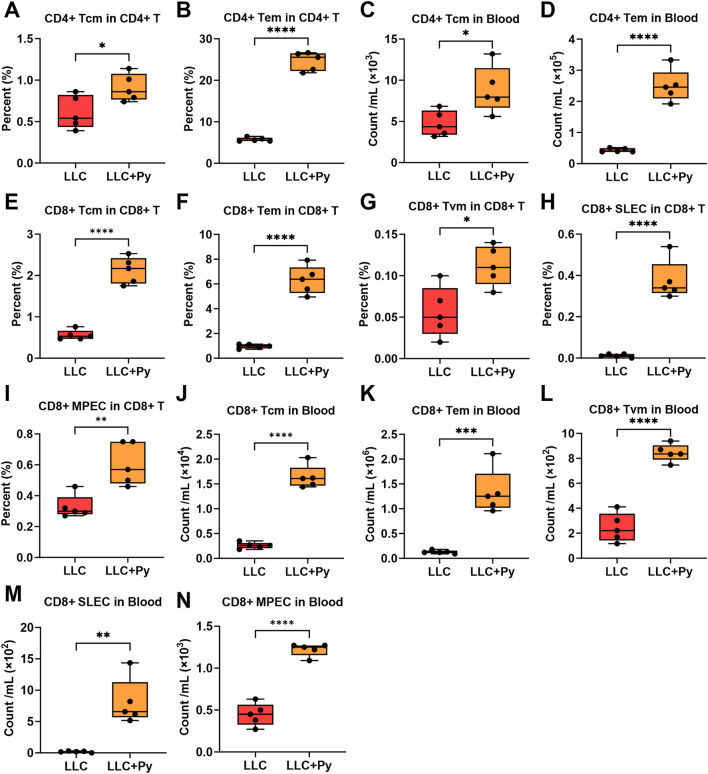
Effect of Py infection on CD4^+^ and CD8^+^ T cell subsets in peripheral blood. **(A)** Percentage of CD4^+^ Tcm in the CD4^+^ T cell population. **(B)** Percentage of CD4^+^ Tem in the CD4^+^ T cell population. **(C)** Count of CD4^+^ Tcm cells per mL peripheral blood. **(D)** Count of CD4^+^ Tem cells per mL peripheral blood. **(E)** Percentage of CD8^+^ Tcm in the CD8^+^ T cell population. **(F)** Percentage of CD8^+^ Tem in the CD8^+^ T cell population. **(G)** Percentage of CD8^+^ Tvm in the CD8^+^ T cell population. **(H)** Percentage of CD8^+^ SLEC in the CD8^+^ T cell population. **(I)** Percentage of CD8^+^ MPEC in the CD8^+^ T cell population. **(J)** Count of CD8^+^ Tcm cells per mL peripheral blood. **(K)** Count of CD8^+^ Tem cells per mL peripheral blood. **(L)** Count of CD8^+^ Tvm cells per mL peripheral blood. **(M)** Count of CD8^+^ SLEC cells per mL peripheral blood. **(N)** Count of CD8^+^ MPEC cells per mL peripheral blood. Data are presented as mean ± SEM (n = 5 per group). Asterisks indicate statistically significant differences (*, *P* < 0.05; **, *P* < 0.01; ***, *P* < 0.001; ****, *P* < 0.0001).

Moreover, Py infection led to a decrease in the percentage of naive CD4^+^ T cells within the CD4^+^ T cell population (*P* < 0.05, Supplementary Figure S5D) and a similar decrease in naive CD8^+^ T cells within the CD8^+^ T cell population (*P* < 0.05, Supplementary Figure S5E). In the aspect of absolute number, Py infection significantly decreased the count of naive CD4^+^ T cells (*P* < 0.05, Supplementary Figure S5F), while increased the count of naive CD8^+^ T cells (*P* < 0.05, Supplementary Figure S5G). Further analysis indicated that Py infection increased the percentages of central memory CD4^+^ T cells (CD4^+^ Tcm) and effector memory CD4^+^ T cells (CD4^+^ Tem) in the total CD4^+^ T cells, and increased the numbers of CD4^+^ Tcm and CD4^+^ Tem in peripheral blood (all *P* < 0.05, Figures 3A–D). Concurrently, Py infection significantly increased the percentages of CD8^+^ Tcm, CD8^+^ Tem, virtual memory CD8^+^ T cells (CD8^+^ Tvm), short-lived effector CD8^+^ T cells (CD8^+^ SLEC), memory precursor CD8^+^ T cells (CD8^+^ MPEC) in the total CD8^+^ T cell population (all *P* < 0.05, Figures 3E–I), and their absolute numbers in peripheral blood (all *P* < 0.05, Figures 3J–N). Overall, Py infection reduced the number of naive CD4^+^ T cells and increased the number of memory CD4^+^ T cells in peripheral blood, indicating that it activated CD4^+^ T cells. At the same time, the infection increased the counts of both naive and memory CD8^+^ T cells in peripheral blood, suggesting that it not only activated CD8^+^ T cells but also promoted the development and maturation of CD8^+^ T cells in the tumor-bearing hosts.

### 3.3 Changes of T cell subsets in tumor tissue

**FIGURE 4:**
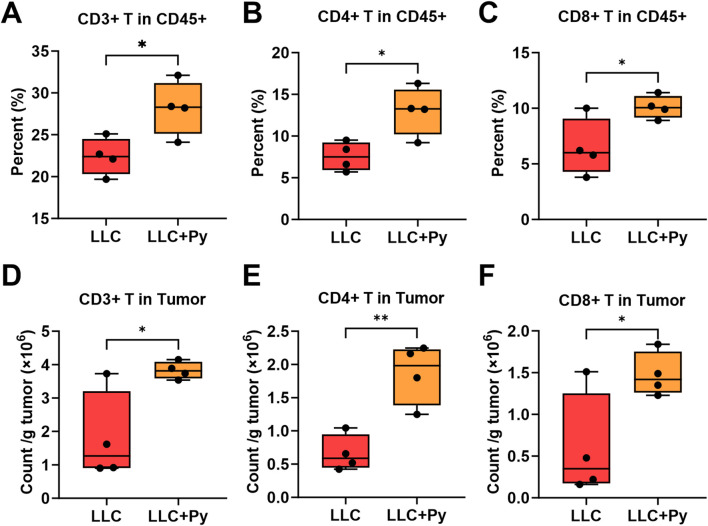
Effect of Py infection on T cell subsets in tumor tissue. **(A)** Percentage of CD3^+^ T cells in the CD45^+^ cell population. **(B)** Percentage of CD4^+^ T cells in the CD45^+^ cell population. **(C)** Percentage of CD8^+^ T cells in the CD45^+^ cell population. **(D)** Count of CD3^+^ T cells per g tumor. **(E)** Count of CD4^+^ T cells per g tumor. **(F)** Count of CD8^+^ T cells per g tumor. Data are presented as mean ± SEM (n = 4 per group). Asterisks indicate statistically significant differences (*, *P* < 0.05; **, *P* < 0.01).

**FIGURE 5:**
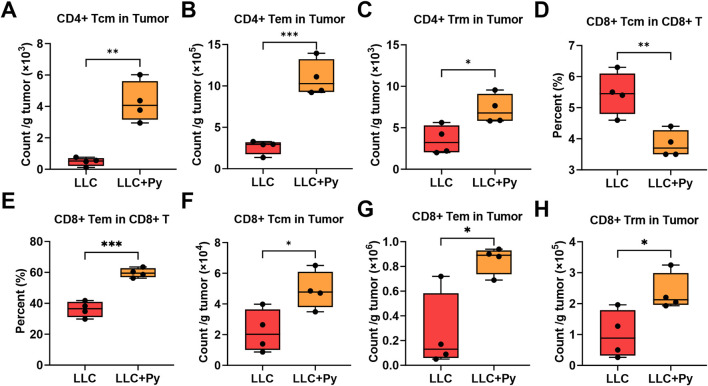
Effect of Py infection on CD4^+^ and CD8^+^ T cell subsets in tumor tissue. **(A)** Count of CD4^+^ Tcm cells per g tumor. **(B)** Count of CD4^+^ Tem cells per g tumor. **(C)** Count of CD4^+^ Trm cells per g tumor. **(D)** Percentage of CD8^+^ Tcm in the CD8^+^ T cell population. **(E)** Percentage of CD8^+^ Tem in the CD8^+^ T cell population. **(F)** Count of CD8^+^ Tcm cells per g tumor. **(G)** Count of CD8^+^ Tem cells per g tumor. **(H)** Count of CD8^+^ Trm cells per g tumor. Data are presented as mean ± SEM (n = 4 per group). Asterisks indicate statistically significant differences (*, *P* < 0.05; **, *P* < 0.01; ***, *P* < 0.001).

To investigate the effects of Py infection on T cell subsets within tumor tissues, high-dimensional flow cytometry was also employed. Py infection did not significantly alter the percentages of CD4^+^ T cells in CD3^+^ T cells (*P* > 0.05, Supplementary Figure S6A), while increased CD8^+^ T in CD3^+^ T cells (*P* < 0.05, Supplementary Figure S6B). Similar to the findings in peripheral blood, Py infection significantly increased the percentages of CD3^+^ T cells, CD4^+^ T cells and CD8^+^ T cells in CD45^+^ cells (all *P* < 0.05, Figures 4A–C), as well as the absolute numbers of CD3^+^ T cells, CD4^+^ T cells and CD8^+^ T cells in the tumor tissues (all *P* < 0.05, Figures 4D–F). In addition, Py infection did not affected the percentages of some CD4^+^ T cell subsets, including naive CD4^+^ T, CD4^+^ Tcm, CD4^+^ Tem in the total CD4^+^ T cells (all *P* > 0.05, Supplementary Figures S6C–E), while decreased the percentage of CD4^+^ tissue resident memory T cells (CD4^+^ Trm) among the total CD4^+^ T cells in tumor tissue (*P* < 0.05, Supplementary Figure S6F). However, Py infection increased the absolute numbers of CD4^+^ Tcm, CD4^+^ Tem, and CD4^+^ Trm (all *P* < 0.05, Figures 5A–C), apart from the naïve CD4^+^ T cells (*P* > 0.05, Supplementary Figure S6G). In the aspect of CD8^+^ T cell subsets, Py infection did not affected the percentages of naïve CD8^+^ T cells in the total CD8^+^ T cells as well as the absolute numbers of CD8^+^ Tvm, and CD8^+^ MPEC cells (all *P* > 0.05, Supplementary Figures S6H–J). Additionally, Py infection decreased the percentages of CD8^+^ Tcm, CD8^+^ Trm, CD8^+^ Tvm, CD8^+^ MPEC in the total CD8^+^ T cells (*P* < 0.05, Figure 5D; Supplementary Figures S6K–M), while increased the percentages and absolute numbers of CD8^+^ Tem and CD8^+^ SLEC cells, and increased the absolute numbers of naïve CD8^+^ T cells, CD8^+^ Tcm and CD8^+^ Trm cells (all *P* < 0.05, Figures 5E–H; Supplementary Figures S6N–P). In particular, CD8^+^ Tem accounted for the majority of CD8^+^ T cells in either group, with a proportion ranging from 40% to 60%; but the treatment group still had a significantly higher proportion than the control group (*P* < 0.05, Figure 5E). On the contrary, in both groups, the proportions of CD8^+^ SLEC among CD8^+^ T cells were the lowest, although the ratio in the treatment group was significantly higher than that in the control group (*P* < 0.05, Supplementary Figure S6O).

### 3.4 Changes of PD-1 expression on T cells in peripheral blood and tumor tissue

**FIGURE 6:**
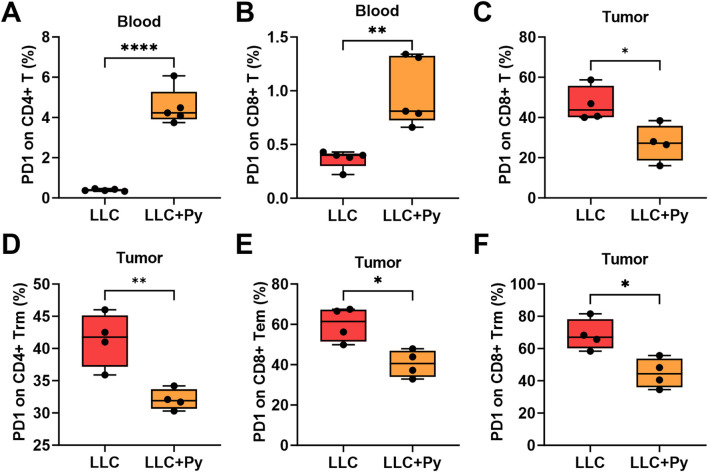
Effect of Py infection on PD-1 expression on T cell subsets in peripheral blood and tumor tissue. **(A)** Percentage of PD-1 expression on CD4^+^ T cells in peripheral blood. **(B)** Percentage of PD-1 expression on CD8^+^ T cells in peripheral blood. **(C)** Percentage of PD-1 expression on CD8^+^ T cells in tumor tissue. **(D)** Percentage of PD-1 expression on CD4^+^ Trm cells in tumor tissue. **(E)** Percentage of PD-1 expression on CD8^+^ Tem cells in tumor tissue. **(F)** Percentage of PD-1 expression on CD8^+^ Trm cells in tumor tissue. Data are presented as mean ± SEM (n = 5 in peripheral blood or n = 4 in tumor each group). Asterisks indicate statistically significant differences (*, *P* < 0.05; **, *P* < 0.01; ***, *P* < 0.001; ****, *P* < 0.0001).

PD-1 (programmed death-1) is a key immune checkpoint molecule and plays a significant role in immunotherapy (Chamoto et al., 2023). Therefore, we analyzed the expressions of PD-1 on T cells and their subtypes in peripheral blood and tumor tissues (Supplementary Figures S7A, B). In peripheral blood, Py infection significantly increased the expressions levels of PD-1 on both CD4^+^ and CD8^+^ T cells (Figures 6A,B). Meanwhile, the expression levels of PD-1 on naïve CD4^+^ and naïve CD8^+^ T cells were relatively low or negligible (even though the level of PD-1 on naïve CD4^+^ T cells in treatment group was significantly higher than that in control group, Supplementary Figures S8A, B). Further analysis revealed that the expression levels of PD-1 were significantly elevated on CD4^+^ Tcm and CD4^+^ Tem (both *P* < 0.05, Supplementary Figures S8C, D). For CD8^+^ T cells subsets, Py infection did not altered PD-1 expressions on CD8^+^ Tcm and CD8^+^ Tem (both *P* > 0.05, Supplementary Figures S8E, F), while increased its expressions on CD8^+^ Tvm, CD8^+^ SLEC and CD8^+^ MPEC (all *P* < 0.05, Supplementary Figures S8G–I).

However, in tumor tissues, Py infection significantly decreased the expression level of PD-1 on CD8^+^ T cells (*P* < 0.05, Figure 6C), while did not change its expression on CD4^+^ T cells (*P* > 0.05, Supplementary Figures S8J). Similar to the situation in peripheral blood, the naïve T cells (both CD4^+^ and CD8^+^) in tumor tissues express extremely low levels or almost undetectable levels of PD-1 in either group (all *P* > 0.05, Supplementary Figures S8K, L). Further analysis revealed that Py infection significantly reduced the expressions of PD-1 on CD4^+^ Trm cells (*P* < 0.05, Figure 6D), CD8^+^ Tem and CD8^+^ Trm cells (both *P* < 0.05, Figures 6E,F), but increased its expression on CD8^+^ Tcm (*P* < 0.05, Supplementary Figure S8M), while did not change its expressions on CD4^+^ Tcm and CD4^+^ Tem, CD8^+^ Tvm, CD8^+^ SLEC and CD8^+^ MPEC cells in tumor tissues (all *P* > 0.05, Supplementary Figures S8N–R). These results indicated that the impacts of Py infection on the expressions of PD-1 on T cells in peripheral blood and tumor tissues were significantly different.

### 3.5 Impact of Py infection on tumor-associated macrophages and myeloid-derived suppressor cells

**FIGURE 7:**
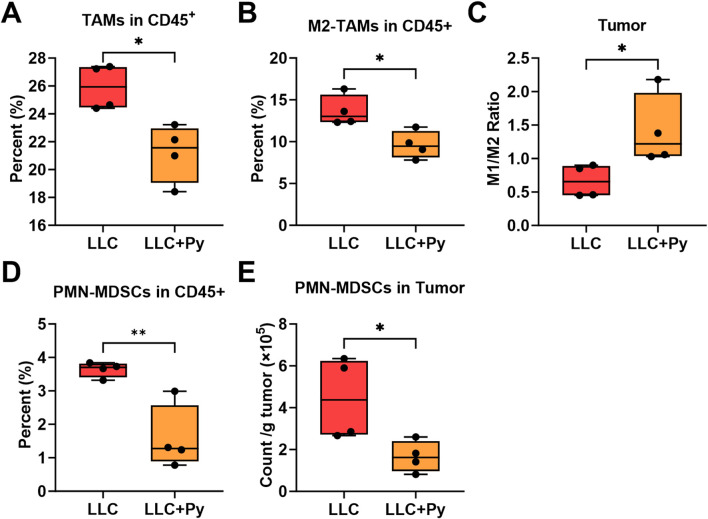
Effect of Py infection on tumor-associated macrophages (TAMs) and myeloid-derived suppressor cells (MDSCs) in tumor tissue. **(A)** Percentage of TAMs among CD45^+^ cells in tumor tissue. **(B)** Percentage of M2-TAMs among CD45^+^ cells in tumor tissue. **(C)** Ratio of M1-TAMs to M2-TAMs in tumor tissue. **(D)** Percentage of polymorphonuclear MDSCs (PMN-MDSCs) among CD45^+^ cells in tumor tissue. **(E)** Count of PMN-MDSCs per g tumor. Data are presented as mean ± SEM (n = 4 each group). Asterisks indicate statistically significant differences (*, *P* < 0.05; **, *P* < 0.01).

Tumor-associated macrophages (TAMs) and myeloid-derived suppressor cells (MDSCs) both play crucial roles in the tumor environment (Ugel et al., 2015). TAMs mainly include antitumor M1-TAMs and protumor M2-TAMs (Kzhyshkowska et al., 2024). MDSCs primarily consist of monocytic MDSCs (M-MDSCs) and polymorphonuclear MDSCs (PMN-MDSCs) (Groth et al., 2019). Therefore, we analyzed TAMs, MDSCs and their subtypes in tumor tissue (Supplementary Figures S9A, B). The results indicated that Py infection significantly decreased the proportions of TAMs (*P* < 0.05, Figures 7A) and the subtype M2-TAMs (*P* < 0.05, Figure 7B) in CD45^+^ cells, while did not change the absolute numbers of TAMs, M1-TAMs, M2-TAMs, and the proportion of M1-TAMs among CD45^+^ cells (all *P* > 0.05, Supplementary Figures S10A–D). In particular, the infection let to an increase in the M1/M2 ratio (*P* < 0.05, Figure 7C), which is consistent with our previous findings in a murine liver cancer model (Wang B. et al., 2020). Furthermore, the infection reduced the proportion of PMN-MDSCs in CD45^+^ cells and absolute number of PMN-MDSCs (all *P* < 0.05, Figures 7D,E), but had no significant impact on the proportions and absolute numbers of MDSCs and the subtype M-MDSCs (all *P* > 0.05, Supplementary Figures S10E–H). These results suggest that Py infection may influence the tumor microenvironment by modulating TAMs polarization, PMN-MDSCs proportion and absolute number, potentially affecting tumor progression.

### 3.6 Py infection reduced the expression levels of pSTAT3 and TGFβ in tumor tissues

**FIGURE 8:**
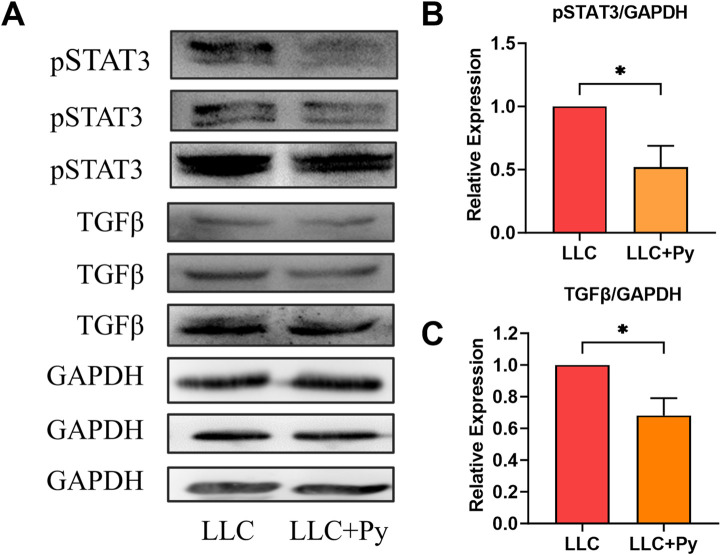
Effect of Py infection on the expressions of pSTAT3 and TGFβ in tumor tissue. On day 19 after tumor inoculation, tumor tissues were harvested for Western blotting. **(A)** The Western blotting results of pSTAT3, TGFβ and GAPDH. **(B)** The relative expression of pSTAT3 in tumor tissues of the LLC group and LLC + Py group (n = 3 per group). The relative expression of pSTAT3 to GAPDH in LLC group were normalized. **(C)** The relative expression of TGFβ in tumor tissues of the LLC group and LLC + Py group (n = 3). The relative expression of TGFβ to GAPDH in LLC group were normalized. GAPDH was used as a loading control for Western blotting. Data are presented as mean ± SEM (n = 3 each group). Asterisks indicate statistically significant differences (*, *P* < 0.05).

STAT3-TGFβ signal pathway plays important roles in the immunosuppressive tumor microenvironment through promoting the infiltration of immunosuppressor cells, such as MDSCs and TAMs in to tumor tissues (Deng et al., 2024; Hu et al., 2020). We detected the expressions of active phosphorylated STAT3 (pSTAT3) and TGFβ in the tumor tissues to reflect the tumor immune microenvironment (Figures 8A). The results indicated that Py infection reduced the expressions of pSTAT3 and TGFβ in the tumor tissues (both *P* < 0.05, Figures 8B,C). These results suggested that Py infection deactivated the tumor immunosuppressive microenvironment through downregulating the expressions of pSTAT3 and TGFβ. Moreover, these phenomena are also consistent with our previous findings (Adah et al., 2019; Chen et al., 2023; Shi et al., 2008).

## 4 Discussion

**FIGURE 9:**
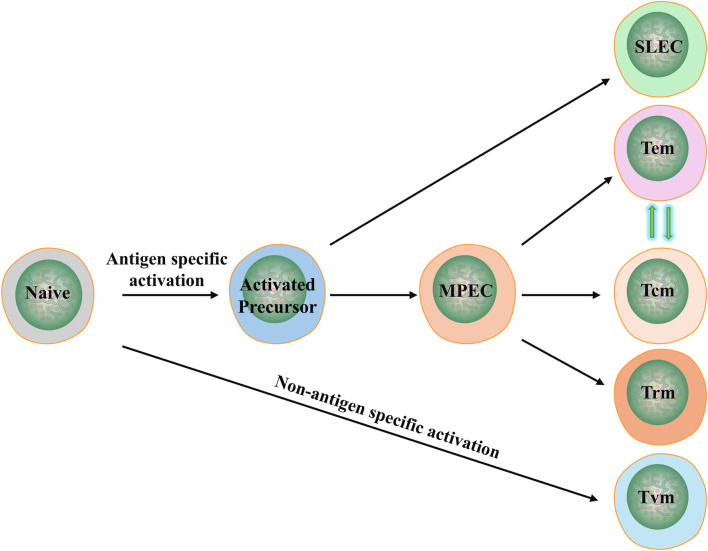
Schematic of activation and differentiation of conventional (α/β) T cells. Naïve: naive T cells; Tcm: central memory T cells; Tem: effector memory T cells; Tvm: virtual memory T cells; Trm: tissue resident memory T cells; SLEC: short-lived effector T cells; MPEC: memory precursor T cells.

In this study, we once again confirmed that *Plasmodium* infection significantly inhibits the growth of lung cancer (LLC) in mice, and for the first time, we used high-dimensional flow cytometry to analyze the pathways through which the infection remodels and activates the immune system. The activation of conventional CD4^+^ and CD8^+^ (α/β) T cells includes two pathways (Figure 9) (Bray et al., 2024). Antigen-specific activation, involves the process from naive T cells to memory precursor effector cells (MPEC), and then to memory cells, namely, Tcm, Tem, Trm and SLEC (Cossarizza et al., 2021; Muroyama and Wherry, 2021) (Schabath and Cote, 2019). Non-antigen-specific activation, that is, from naive T cells to Tvm (Seok et al., 2023). Among them, Tcm and Tem usually undergo mutual transformation. Of course, all these memory cells can transform under certain conditions (see the following description for details). Current study indicates that after *Plasmodium* infection, although the proportions of activated T cells and their subpopulations in peripheral blood and tumor tissues show considerable inconsistency, their absolute counts are consistently and significantly increased in both peripheral blood and tumor tissues. For instance, in peripheral blood, the absolute counts of CD3^+^ T cells, CD4^+^ and CD8^+^ T cells, CD4^+^ Tcm, CD4^+^ Tem, CD8^+^ naïve, CD8^+^ Tcm, CD8^+^ Tem, CD8^+^ Tvm, CD8^+^ SLEC and CD8^+^ MPEC are all significantly upregulated; while in tumor tissues, the absolute counts of CD3^+^ T cells, CD4^+^ and CD8^+^ T cells, CD4^+^ Tcm, CD4^+^ Tem, CD4^+^ Trm, CD8^+^ naïve, CD8^+^ Tcm, CD8^+^ Tem, CD8^+^ Trm, and CD8^+^ SLEC are also significantly upregulated (see Supplementary Table S3).

Tcm cells possess a high capacity for self-renewal and proliferation. Upon detecting antigenic threats, Tcm cells can rapidly differentiate into effector T cells, providing a quick and effective immune response (Liu et al., 2020). In tumors, they can provide a sustained immune response and contribute to the generation of long-lived memory T cells. Their presence in tumor tissues is associated with better clinical outcomes in some cancer types, such as non-small cell lung cancer (NSCLC), where higher frequencies of Tcm cells in tumor-infiltrating lymphocytes are linked to improved patient survival (Wang et al., 2021). Py infection increases the absolute numbers of both CD4^+^ Tcm and CD8^+^ Tcm cells in peripheral blood and tumor tissues, thereby enhancing their capacity to eliminate tumor cells. Tem cells are the primary effectors of the memory T cell response and the first line of defense against pathogens, and play a critical role in providing long-lived immunity against various pathogens (Liu et al., 2020). They can rapidly produce effector cytokines such as IFN-γ and TNF-α upon antigen re-exposure and exert cytotoxic effects to eliminate infected cells and tumor cells. The proportion of Tem cells in tumor tissues varies depending on factors such as the tumor type and stage (Wang et al., 2021). In some tumors, Tem cells are the predominant T cell subset (Wang et al., 2021). CD8^+^ Tem cells, with their highly efficient effector functions, play a crucial role in antitumor responses (Tiberti et al., 2022). In our current study, Py infection significantly upregulates the percentages and absolute numbers of CD4^+^ Tem and CD8^+^ Tem in both peripheral blood and tumor environment, suggesting an enhancement of the antitumor immunity. Trm cells can recognize tumor-associated antigens and exert antitumor effects through direct cytotoxicity, the release of cytokines and activation of other immune cells (Park et al., 2019; Gavil et al., 2024). Trm cells can provide immediate and potent local immune responses against tumors. Accumulating evidence from multiple human cancer studies, including melanoma (Edwards et al., 2018; Malik et al., 2017; Molodtsov et al., 2021), breast cancer (Savas et al., 2018; Losurdo et al., 2021), and lung cancer (Ganesan et al., 2017; Djenidi et al., 2015), indicates a positive correlation between Trm cell infiltration and improved patient survival. Notably, our current study reveals that Py infection increases the absolute numbers of CD4^+^ Trm and CD8^+^ Trm cells in tumor tissues. Moreover, Trm cells play a role in suppressing dormant micro-metastases, thereby reducing the recurrence and spread of the tumor (Molodtsov et al., 2021; Tallon de Lara et al., 2021). Tvm cells are a unique antigen-inexperienced subset of CD8^+^ T cells with a high degree of plasticity (Viano et al., 2022). Their plasticity allows them to adapt to clonally expand and induce the occurrence of Trm cells within the tumor, and play a role in immune surveillance and antitumor immunity (Hou et al., 2021). CD8^+^ Tvm cells have a relatively small percentage of total CD8^+^ T cells in peripheral blood, while had a higher percentage in tumor tissues, which is consistent with our current findings. SLEC cells act as a “rapid response force” in tumor immunity, capable of quickly recognizing and directly killing tumor cells expressing specific antigens, through secreting cytokines such as IFNγ and TNFα to enhance antitumor immune responses (Muroyama and Wherry, 2021; Gebhardt et al., 2023). However, due to their short lifespan and susceptibility to exhaustion under persistent antigen stimulation, they need to maintain a balance with other T cell subgroups to achieve both rapid and sustained antitumor effects (Muroyama and Wherry, 2021; Gebhardt et al., 2023). Our current results indicates that Py infection increases the percentage and absolute number of CD8^+^ SLEC in both peripheral blood and tumor tissues, suggesting an enhancement of antitumor immunity. The main function of MPEC is to patrol in the peripheral blood, monitor pathogen invasion, then generate long-lived memory T cells and sustain antitumor immune responses once they detect antigen signals (Muroyama and Wherry, 2021; O'Sullivan et al., 2023). In peripheral blood, MPEC cells usually account for a relatively small proportion among CD8^+^ T cells. However, in tumor tissues, the proportion of MPEC cells varies depending on factors such as tumor type and individual patient differences (Muroyama and Wherry, 2021; O'Sullivan et al., 2023). When MPEC cells are exposed to strong stimulation signals, they may further differentiate into other memory T cells. This might explain our current observations: *Plasmodium* infection increases the ratio and absolute count of MPEC in the peripheral blood, but does not change the absolute number of MPEC in the tumor tissues.

Similar to our previous research results, Py infection promotes the infiltration of activated T cells into tumor tissues (Chen et al., 2011; Tao et al., 2022). However, in the current study, we observed for the first time that multiple subtypes of activated T cells enter the tumor tissues, which should be related to the simultaneous relief of the immunosuppressive tumor microenvironment by the infection. Our previous research on mouse lung cancer model indicates that Py infection significantly downregulates the numbers and functions of MDSCs and Tregs in tumor tissues through inhibiting the secretion of a series of cytokines and chemokines by cancer cells, thereby facilitating the entry of antitumor immune cells into tumor tissues, and stimulating their antitumor effects, such as down-regulating the PD-1 expression level of CD8^+^ T cells and promoting their secretion of antitumor effector molecules perforin and granzyme B. Moreover, the functional molecules that play the roles may exist in *Plasmodium*-associated exosomes (Adah et al., 2019). In current study, we also found that the infection significantly reduces the number of PMN-MDSCs in tumor tissues. In previous study on a mouse liver cancer model, we discovered that Py infection significantly decreases the number of TAMs, while significantly increasing the ratio of M1/M2 macrophages in tumor tissues (Wang B. et al., 2020). Moreover, the results indicated that these effects are closely related to the metabolite of *Plasmodium* parasites, namely, malaria pigment hemozoin (Wang B. et al., 2020). In current study, we further confirmed in the mouse lung cancer model that the infection reduces the number of TAMs and alters the polarization of TAMs, that is, it increases the ratio of M1/M2. Thus, it is conducive to the occurrence of antitumor immune responses. This might also be one of the reasons for the significant downregulation of PD-1 expression levels of CD4^+^ and CD8^+^ T cells observed in the tumor tissues in this study. But interestingly and importantly, the level of PD-1 on T cells in the peripheral blood not only does not decrease, but instead significantly increases. This is similar to the phenomenon we observed in our previous study in a murine triple-negative breast cancer (4T1) model (Pan et al., 2021). This partly explains why, in mice infected with the parasites, there are high levels of cytokines in the body, but no occurrence of the cytokine release syndrome as seen in CAR-T cell therapy (Cosenza et al., 2021). Of course, we cannot therefore conclude that *Plasmodium* infection can directly activate the tumor-specific T cell response. This is because in our previous research, it has been demonstrated that the infection promotes the maturation of dendritic cells (DCs), and promotes the influx of mature DCs into the draining lymph nodes surrounding the tumor (Chen et al., 2011). Therefore, we can consider that *Plasmodium* infection activates the tumor-specific T cells through the action of antigen-presenting cells such as DCs. However, before that, *Plasmodium* infection first activates innate immune cells, such as NK cells (as shown in our past research) and Tvm (as shown in our current study), and promotes the infiltration of these cells into tumor tissues. After these innate immune cells enter the tumor tissues, they kill some tumor cells, and the dead tumor cells would release tumor antigens and tumor-associated antigens. Under the condition of high levels of Th1 type cytokines during *Plasmodium* infection (Stevenson and Riley, 2004), these antigens activate DCs, and then activate tumor antigen-specific T cells. Our previous studies have strongly demonstrated that *Plasmodium* infection can activate tumor-specific immunity. For instance, the infection can cure approximately 5%–10% (1–2 per 20 mice) of mouse lung cancer or, when combined with radiotherapy, can cure 70% of mouse brain glioma. Moreover, when these cured mice are inoculated with the same type of tumor cells, no tumor formation occurs, but when inoculated with a different type of tumor cells, tumor formation does occur. This indicates that these cured mice retain long-lasting tumor-specific immune memory (Chen et al., 2011; Tao et al., 2022). Our current study also suggests that *Plasmodium* infection not only activates T cells, but may also promote the development and maturation of CD8^+^ T cells, because the numbers of naive CD8^+^ T cells significantly increased both in the peripheral blood and tumor tissues of the infected mice. This result is basically consistent with the findings of our previous study on macaque model, namely, that *Plasmodium* infection significantly increases the numbers of naive CD4^+^ and CD8^+^ T cells as well as memory CD4^+^ and CD8^+^ T cells in the peripheral blood. Moreover, the quantities of these cells remain significantly higher than those before infection for 5 months after termination of the infection (Li et al., 2012).

We further analyzed the expression levels of pSTAT3 and TGFβ in tumor tissues. The results show that *Plasmodium* infection significantly downregulates the expression of these two signaling molecules (Figure 8), providing direct evidence that the infection activates the antitumor immune responses. The expression and activation of STAT3 (in its phosphorylated form, pSTAT3) are associated with immunosuppression. The function of TGFβ is similar to that of pSTAT3, with a strong immunosuppressive effect, and the two are mutually upstream and downstream signaling molecules (Hu et al., 2020; Yu et al., 2009). Therefore, their expressions can form a vicious cycle. *Plasmodium* infection inhibits the expressions of these two signaling molecules in tumor tissues, precisely explaining the reductions in the numbers of PMN-MDSCs and TAMs, the expression level of PD-1 on T cells, and the increase in the ratio of M1/M2 in tumor tissues. At the same time, pSTAT3 is also related to tumor angiogenesis (Hu et al., 2020). Our previous research has shown that exosomes isolated from the plasma of mice infected with *Plasmodium* parasites can inhibit tumor angiogenesis. Further analysis revealed that these exosomes contain at least four microRNAs, namely, miRNA 16/322/497/17 (Yang et al., 2017). Meanwhile, we discovered a new long non-coding RNA in the tumor tissues of *Plasmodium*-infected mice, named lncRNA F66. All these RNAs target the VEGFR2 gene in tumor vascular endothelial cells, preventing its expression and thus inhibiting tumor angiogenesis (Qin et al., 2020; Yang et al., 2017). Hemozoin, a metabolite of *Plasmodium* parasites, also inhibits tumor angiogenesis by blocking the IGF-1/MMP9 signaling pathway in TAMs (Wang B. et al., 2020). In addition, TGFβ can mediates epithelial-mesenchymal transition (EMT) and tumor metastasis (Hao et al., 2019). We also found that *Plasmodium* infection blocks the TGFβ/CCR10/PI3K/Akt/GSK-3β signaling pathway, thereby inhibiting the EMT of cancer cells, and therefore preventing tumor recurrence and metastasis (Liang et al., 2021; Chen et al., 2023). Further systematic analysis also revealed that the expressions of pSTAT3 and TGFβ are associated with 14 cancer hallmarks in tumor occurrence and development (Wang H. Q. et al., 2020; Mortezaee and Majidpoor, 2022). One (XC) of us recently established a novel theory called immunodynamics/tumor ecodynamics (Chen, 2022; Chen, 2024), which provides a completely new perspective to explain the mechanisms of action of *Plasmodium* infection against cancer, and summarizes the 14 cancer hallmarks as the 14 functional phenotypes of the complex tumor ecosystem (Chen, 2024). Therefore, it can be concluded that *Plasmodium* infection targets and inhibits the entire tumor ecosystem by suppressing the pSTAT3 and TGFβ signals. In principle, this is different from the currently widely used single-target immune checkpoint blockade therapies in clinical practice (Meng et al., 2024). The latter can only reactivate T cells, especially CD8^+^ T cells, which were once activated by cancer cells but are then suppressed by them, and it requires that these T cells exist in the tumor tissues before treatment (hot tumors) for the therapies to be effective. If these T cells do not exist in the tumor tissues before treatment (cold tumors), there will be no therapeutic effect or the effect will be limited. For instance, immune checkpoint blockade therapy alone has no or limited efficacy on cold tumors such as brain glioma (Wang et al., 2023). However, *Plasmodium* immunotherapy (benign form of *Plasmodium* infection) has therapeutic effects on both hot tumors (such as Lewis lung cancer) and cold tumors (such as glioma). Therefore, we have reasons to believe that both cold and hot tumors can be treated first with *Plasmodium* immunotherapy, converting the cold into the hot or making hot even hotter; then, the immune checkpoint blockade therapy can be used for the subsequent treatment. This sequential regimen should become a new mode for cancer treatment in the future. Due to the fact that *Plasmodium* immunotherapy can fully activate the immune system and suppress the entire tumor ecosystem, it is also called immune ecotherapy or systemic immunotherapy (Chen, 2024), a completely new type of immunotherapy.

The limitation of this study is the lacks of *ex vivo* experiments to detect the function of cells responsible for cellular immune responses.

Based on our previous researches, several clinical studies on *Plasmodium* immunotherapy/immune ecotherapy for the treatment of advanced cancers are underway in China (NCT02786589, NCT3375983, NCT03474822, NCT03375983, NCT05924776). It is expected that the clinical research results will be published in the near future.

## 5 Conclusion

Based on our past and current studies, we can draw the following conclusion: *Plasmodium* infection comprehensively remodels and activates the immune system, and targets/inhibits the entire tumor ecosystem by downregulating pSTAT3 and TGFβ signals.

## Data Availability

The original contributions presented in the study are included in the article/Supplementary Material, further inquiries can be directed to the corresponding authors.
