# Supplementary material for: Plasmodium infection fully activates the immune system in peripheral blood and tumor microenvironment in a murine Lewis lung cancer model
Source: Front Mol Biosci. 2026 Jan 28;12:1724792. doi: 10.3389/fmolb.2025.1724792 (PMC12892102; doi:10.3389/fmolb.2025.1724792)
Supplement: Supplementary file 1 [file Supplementaryfile1.docx]

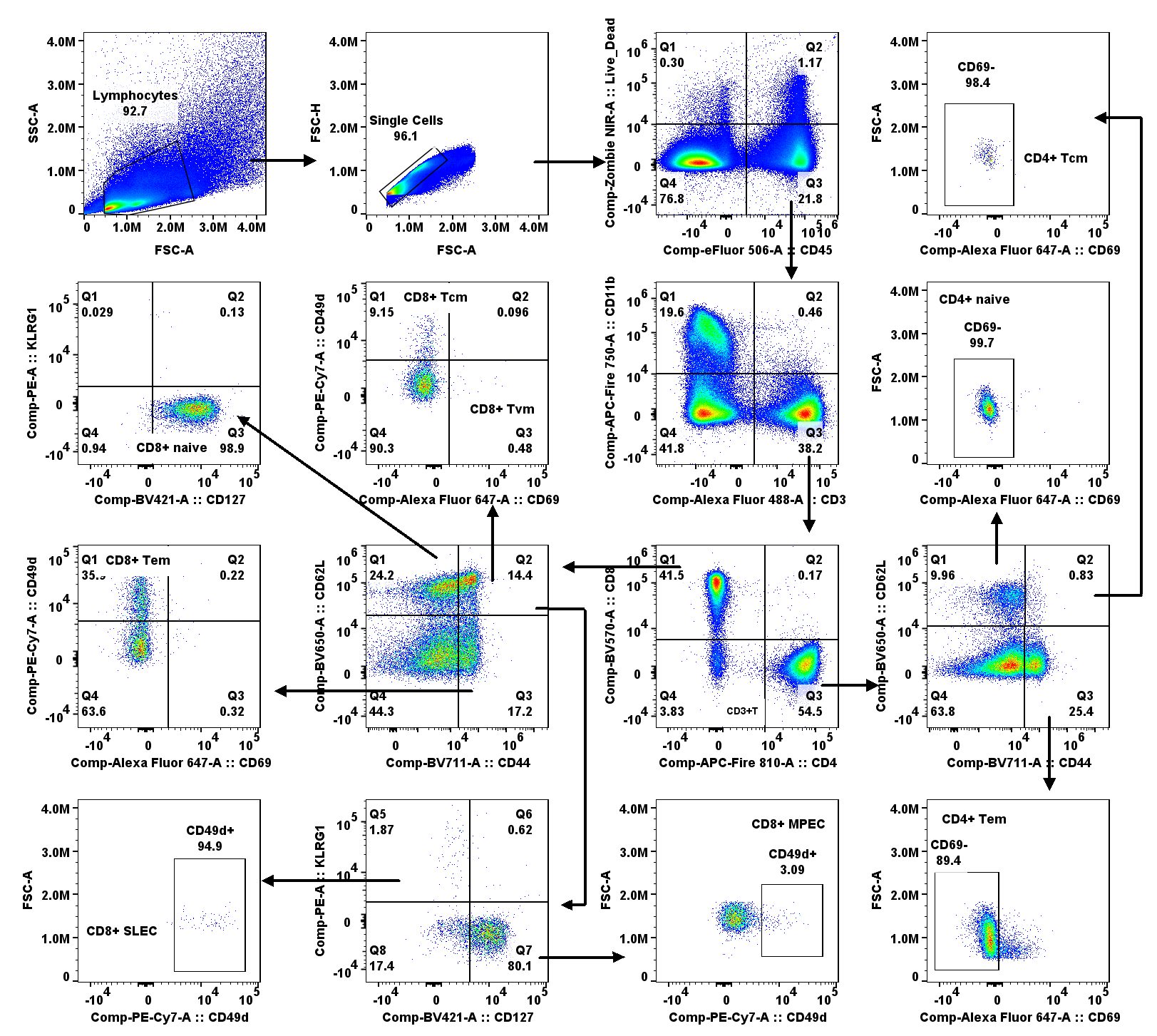


**Supplementary Figure 1.** Representative flow cytometry strategies for gating T cell and its subsets in peripheral blood. Naïve: naive T cell, Tcm: central memory T cell, Tem: effector memory T cell, Tvm: virtual memory T cell, SLEC: short-lived effector T cell, MPEC: memory precursor T cell.


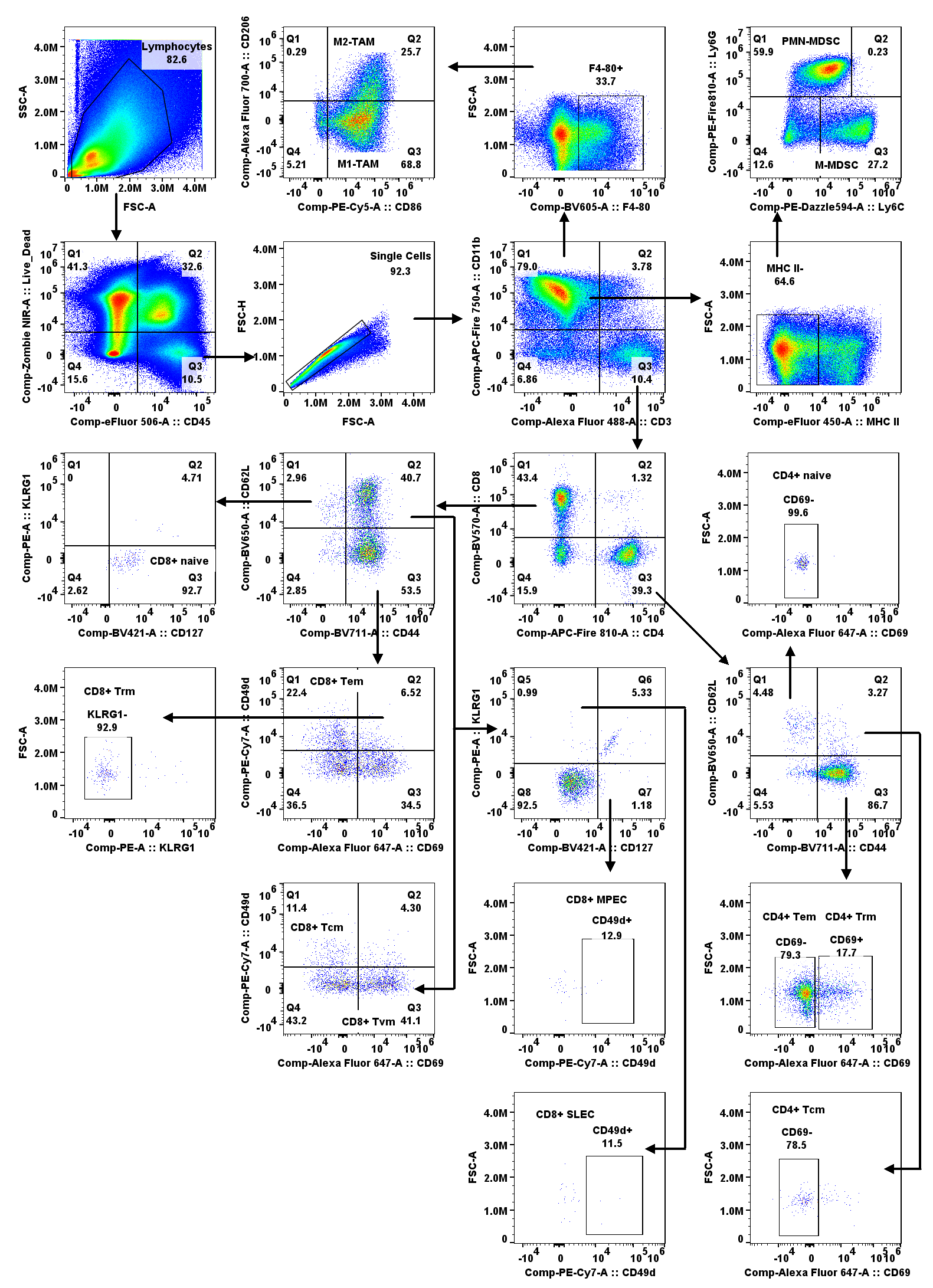


**Supplementary Figure 2.** Representative flow cytometry strategies for gating immune cells in tumor tissue. Naïve: naïve T cell; Tcm: central memory T cell; Tem: effector memory T cell; Tvm: virtual memory T cell; Trm: tissue resident memory T cell; SLEC: short-lived effector T cell; MPEC: memory precursor T cell; M1-TAM: M1 tumor-associated macrophage; M2-TAM: M2 tumor-associated macrophage; M-MDSC: monocytic myeloid-derived suppressor cell; PMN-MNDSC: polymorphonuclear myeloid-derived suppressor cell.


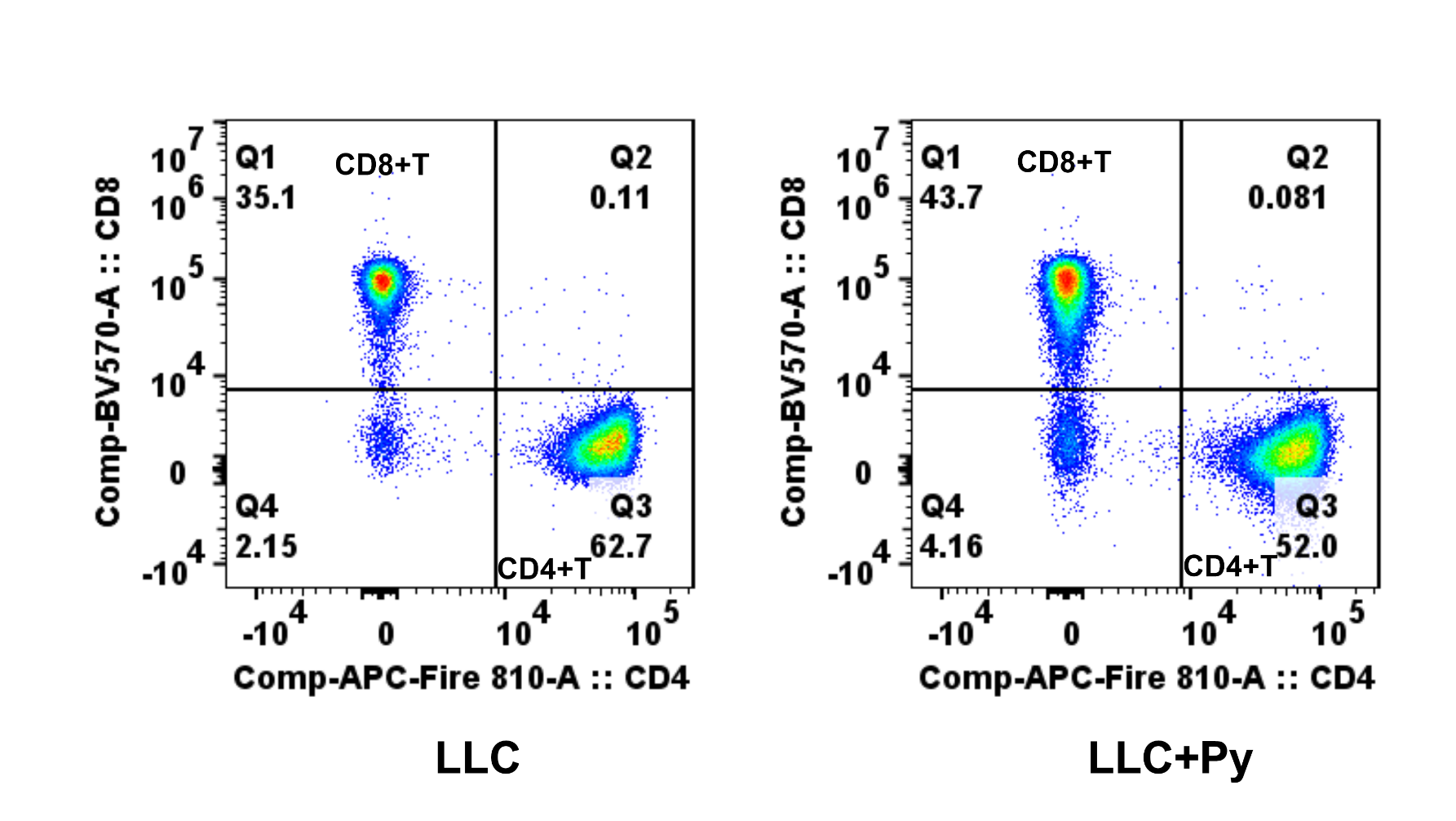


**Supplementary Figure 3.** Representative flow cytometry plot for gating CD4+ and CD8+ T cells in CD3+ T cell population in peripheral blood.


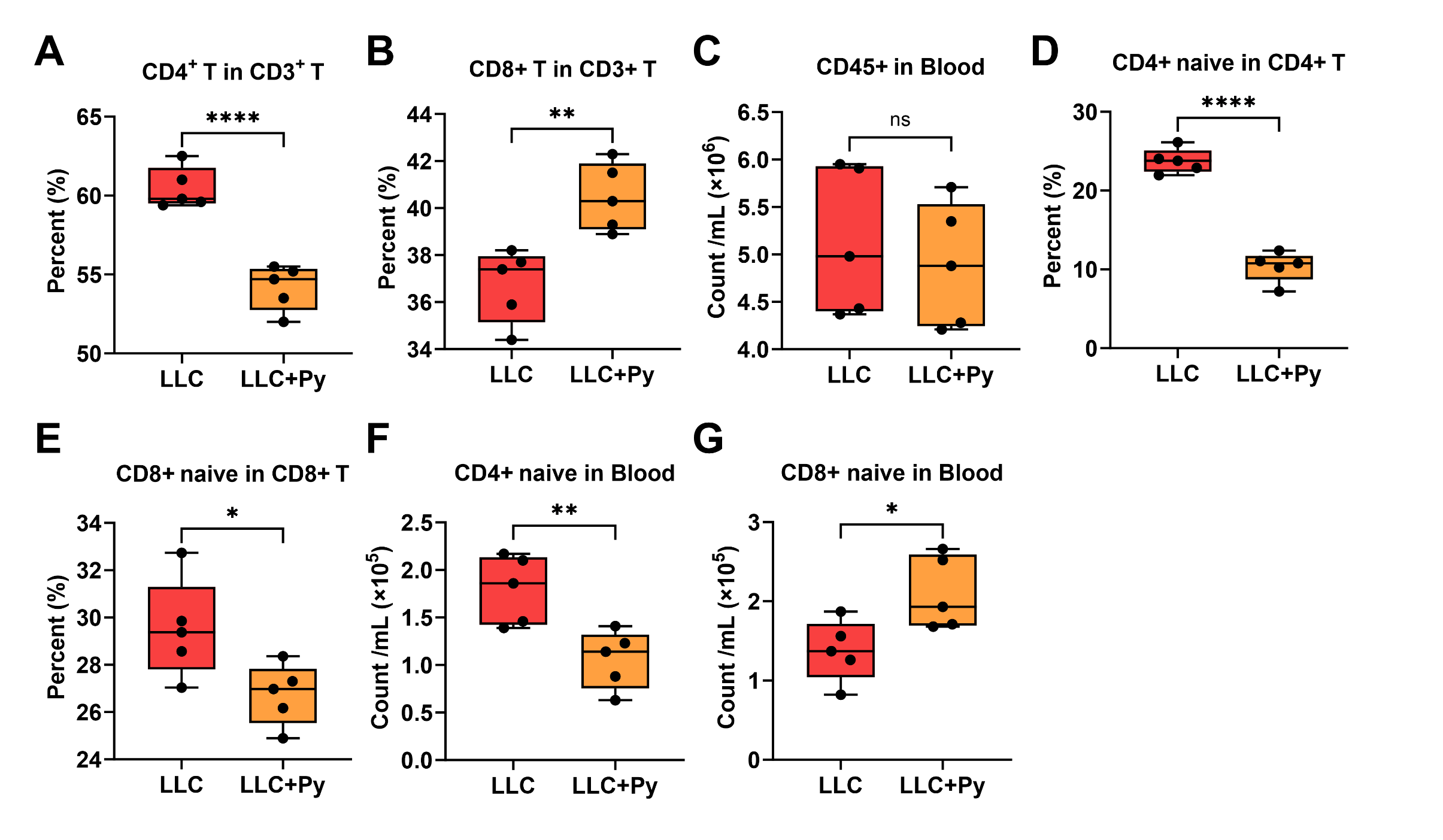


**Supplementary Figure** **4.** Effect of Py infection on T cells in peripheral blood. **(A)** Percentage of CD4+ T cells in the CD3+ T cell population. **(B)** Percentage of CD8+ T cells in the CD3+ T cell population. **(C)** Count of CD45+ cells per mL peripheral blood. **(D)** Percentage of naïve CD4+ T cells in the CD4+ T cell population. **(E)** Percentage of naïve CD8+ T cells in the CD8+ T cell population. **(F)** Count of naïve CD4+ T cells per mL peripheral blood. **(G)** Count of naïve CD8+ T cells per mL peripheral blood. Data are presented as mean ± SEM (n = 5 per group). "ns" indicates no statistically significant difference, while asterisks indicate statistically significant differences (*, *P* < 0.05; **, *P* < 0.01; ***, *P* < 0.001; ****, *P* < 0.0001).


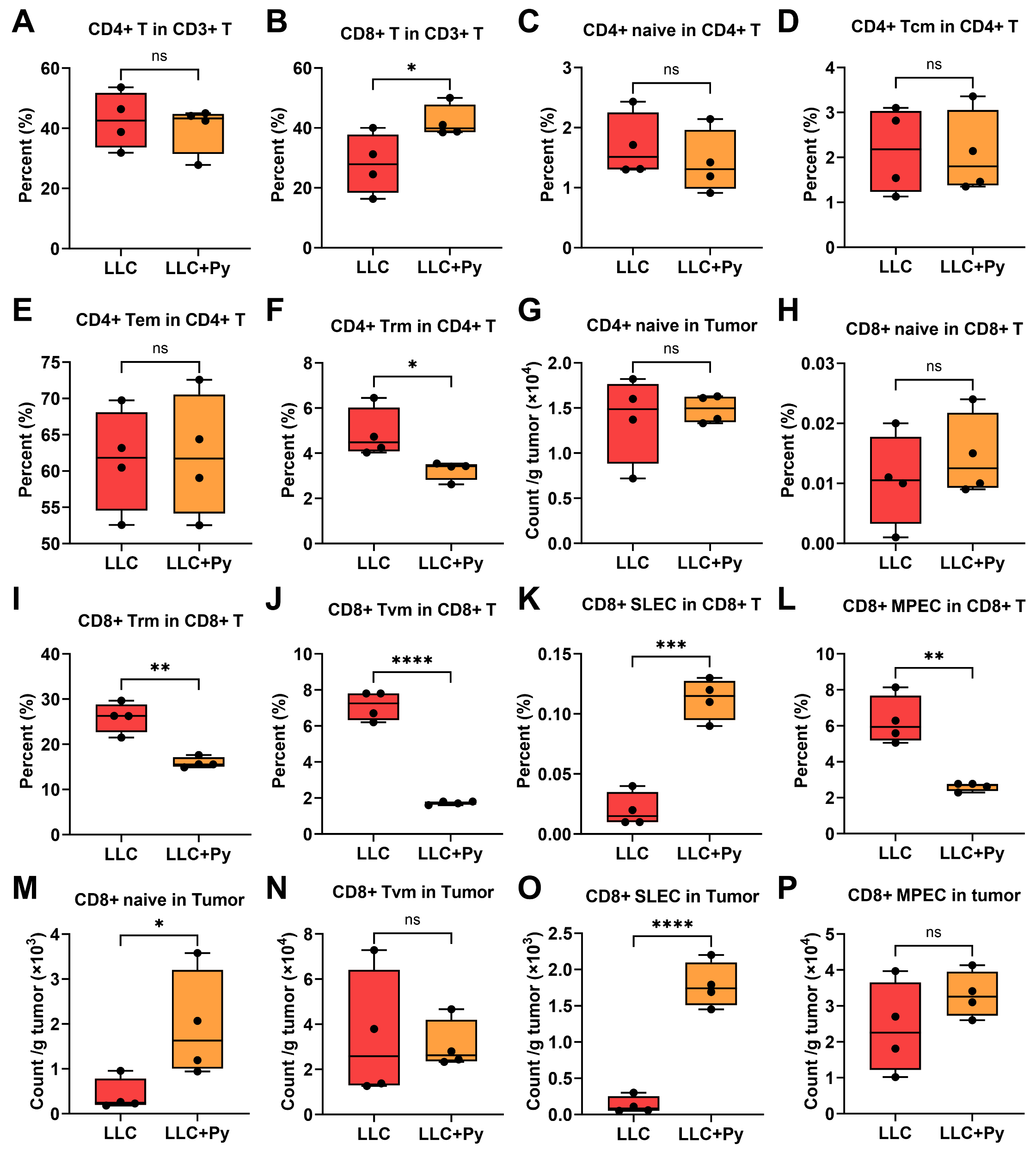


**Supplementary Figure** **5.** Effect of Py infection on CD4+ and CD8+ T cell subsets in tumor tissue. **(A)** Percentage of CD4+ T cells in the CD3+ T cell population. **(B)** Percentage of CD8+ T cells in the CD3+ T cell population. **(C)** Percentage of naïve CD4+ T cells in the CD4+ T cell population. **(D)** Percentage of CD4+ Tcm cells in the CD4+ T cell population. **(E)** Percentage of CD4+ Tem cells in the CD4+ T cell population. **(F)** Percentage of CD4+ Trm cells in the CD4+ T cell population. **(G)** Count of naïve CD4+ T cells per g tumor. **(H)** Percentage of naïve CD8+ T cells in the CD8+ T cell population. **(I)** Percentage of CD8+ Trm cells in the CD8+ T cell population. **(J)** Percentage of CD8+ Tvm cells in the CD8+ T cell population. **(K)** Percentage of CD8+ SLEC cells in the CD8+ T cell population. **(L)** Percentage of CD8+ MPEC cells in the CD8+ T cell population. **(M)** Count of naïve CD8+ T cells per g tumor. **(N)** Count of CD8+ Tvm cells per g tumor. **(O)** Count of CD8+ SLEC cells per g tumor. **(P)** Count of CD8+ MPEC cells per g tumor. Data are presented as mean ± SEM (n = 4 per group). "ns" indicates no statistically significant difference, while asterisks indicate statistically (*, *P* < 0.05; **, *P* < 0.01; ***, *P* < 0.001; ****, *P* < 0.0001).


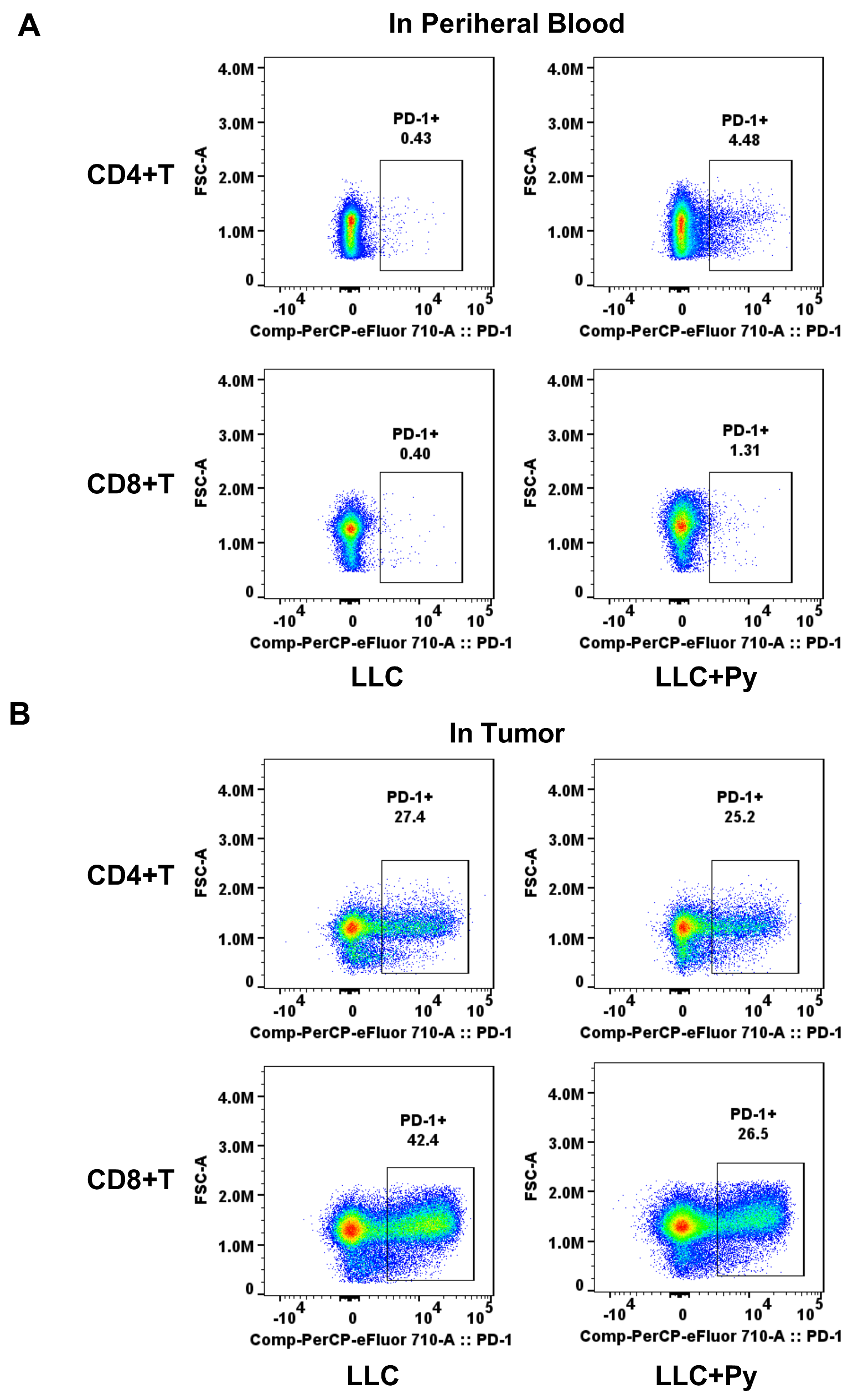


**Supplementary Figure 6.** Representative flow cytometry plot for gating PD-1 expressions on CD4+ T cells and CD8+ T cells. **(A)** Representative flow cytometry plot for gating PD-1 expressions on CD4+ T cells and CD8+ T cells in peripheral blood. **(B)** Representative flow cytometry plot for gating PD-1 expressions on CD4+ T cells and CD8+ T cells in tumor tissue.


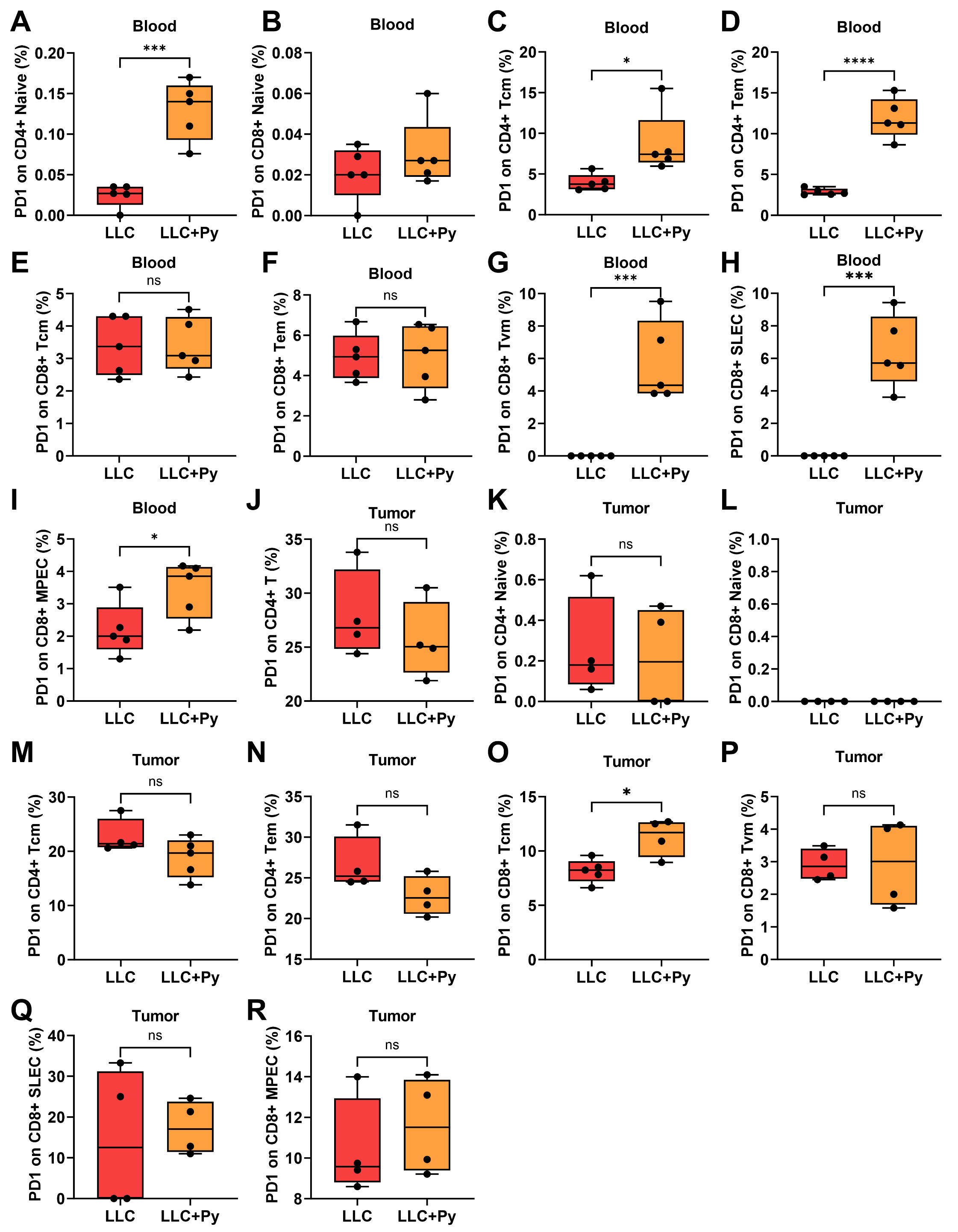


**Supplementary Figure 7.** Effect of Py infection on PD-1 expressions on T Cell subsets in peripheral blood and tumor tissue. **(A)** Percentage of PD-1 expression on naïve CD4+ T cells in peripheral blood. **(B)** Percentage of PD-1 expression on naïve CD8+ T cells in peripheral blood. **(C)** Percentage of PD-1 expression on CD4+ Tcm in peripheral blood. **(D)** Percentage of PD-1 expression on CD4+ Tem in peripheral blood. **(E)** Percentage of PD-1 expression on CD8+ Tcm in peripheral blood. **(F)** Percentage of PD-1 expression on CD8+ Tem in peripheral blood. **(G)** Percentage of PD-1 expression on CD8+ Tvm in peripheral blood. **(H)** Percentage of PD-1 expression on CD8+ SLEC in peripheral blood. **(I)** Percentage of PD-1 expression on CD8+ MPEC in peripheral blood. **(J)** Percentage of PD-1 expression on CD4+ T cells in tumor tissue. **(K)** Percentage of PD-1 expression on naïve CD4+ T cells in tumor tissue. **(L)** Percentage of PD-1 expression on naïve CD8+ T cells in tumor tissue. **(M)** Percentage of PD-1 expression on CD4+ Tcm in tumor tissue. **(N)** Percentage of PD-1 expression on CD4+ Tem in tumor tissue. **(O)** Percentage of PD-1 expression on CD8+ Tcm in tumor tissue. **(P)** Percentage of PD-1 expression on CD8+ Tvm in tumor tissue. **(Q)** Percentage of PD-1 expression on CD8+ SLEC in tumor tissue. **(R)** Percentage of PD-1 expression on CD8+ MPEC in tumor tissue. Data are presented as mean ± SEM (n = 5 per group in peripheral or n = 4 per group in tumor tissue). "ns" indicates no statistically significant difference, while asterisks indicate statistically significant difference (*, *P* < 0.05; **, *P* < 0.01; ***, *P* < 0.001; ****, *P* < 0.0001).


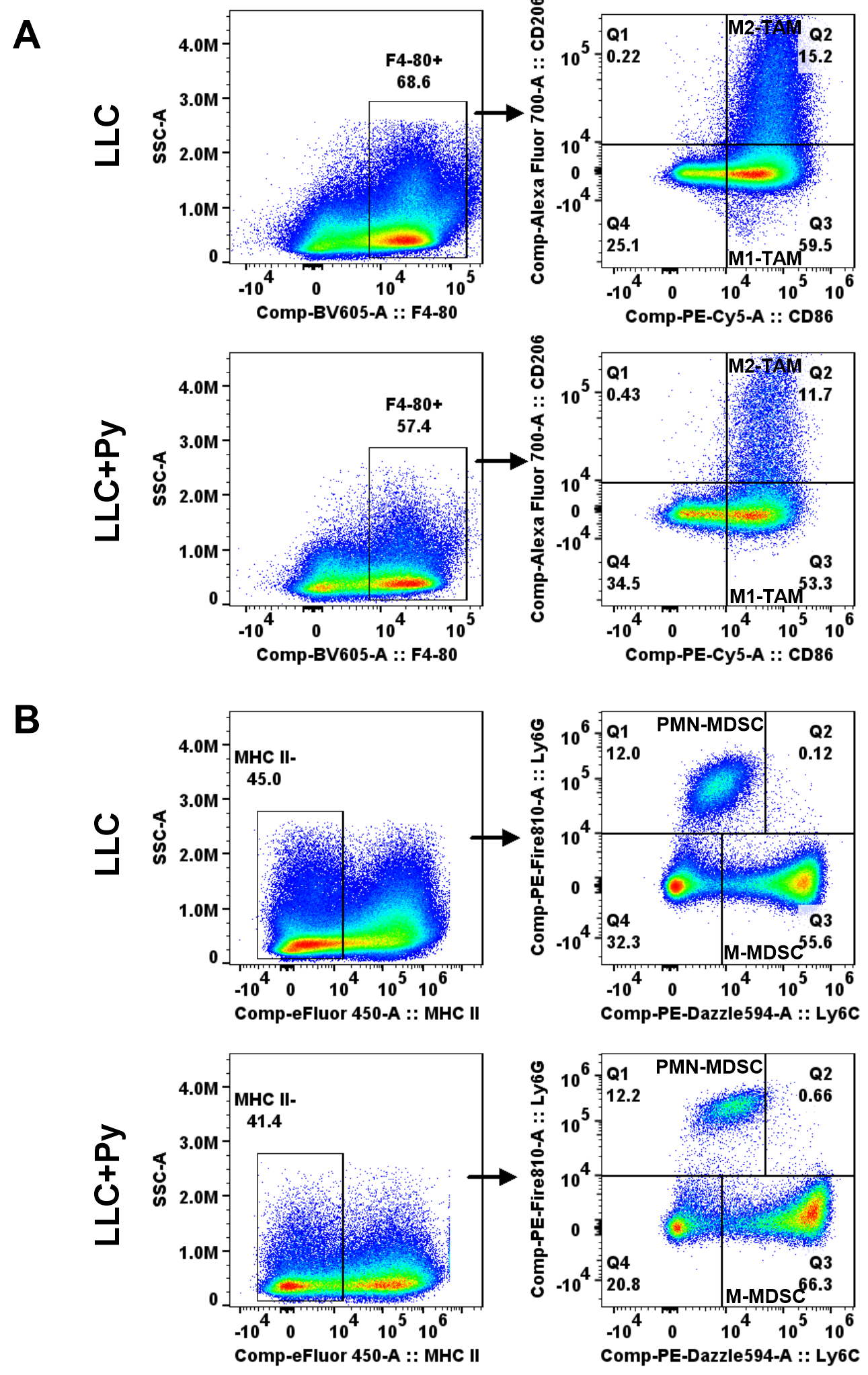


**Supplementary Figure 8.** Representative flow cytometry plot for gating tumor-associated macrophage (TAM) and myeloid-derived suppressor cell (MDSC) population. **(A)** Representative flow cytometry plot for gating M1-TAM and M2-TAM cell populations in tumor tissue for LLC group and LLC+Py group. **(B)** Representative flow cytometry plot for gating monocytic MDSC (M-MDSC) and polymorphonuclear MDSC (PMN-MDSC) populations in tumor tissue for LLC group and LLC+Py group.


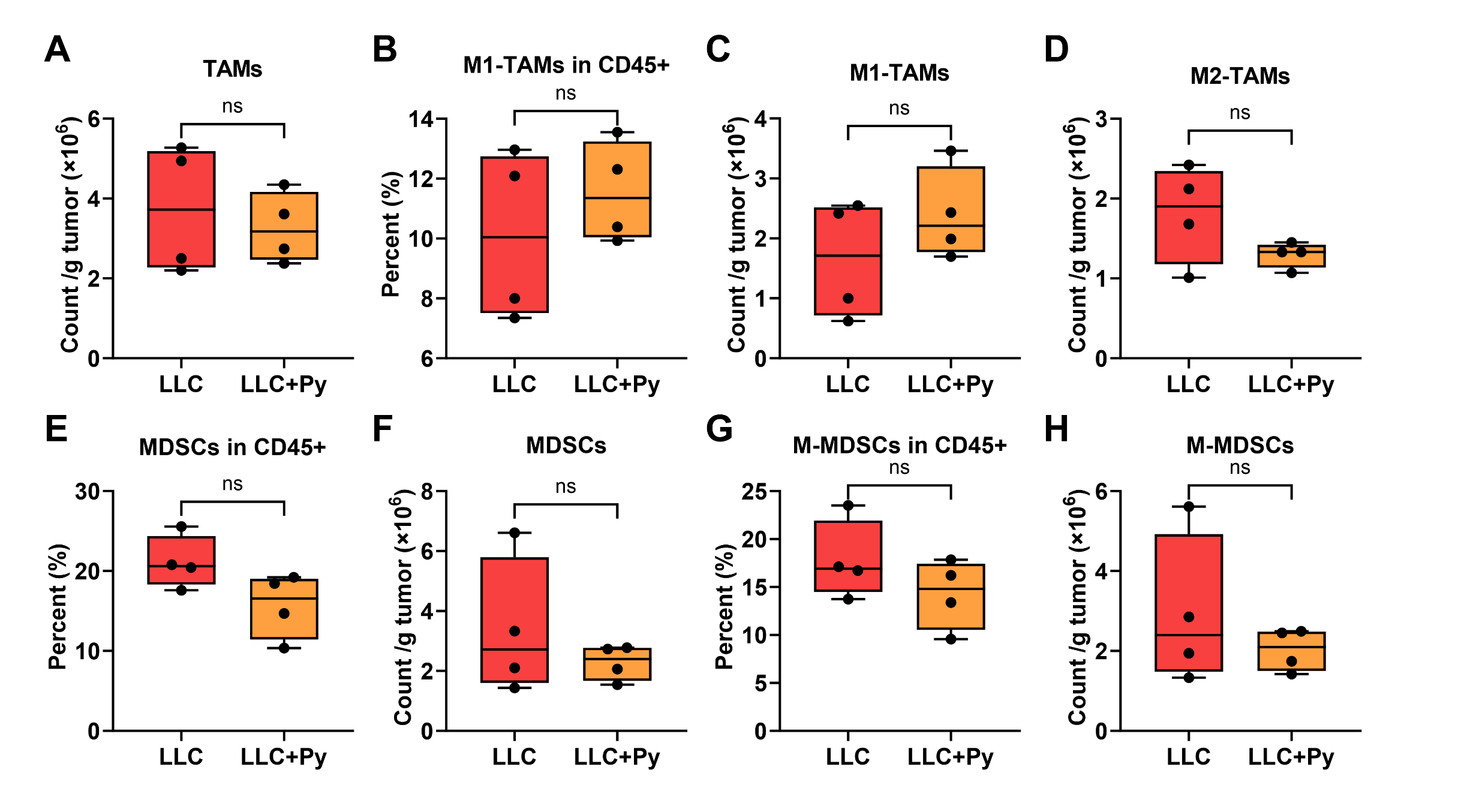


**Supplementary Figure** **9.** Effect of Py infection on TAMs and MDSCs in tumor tissue. **(A)** Count of TAMs among CD45+ cells in tumor tissue. **(B)** Percentage of M1-TAMs among CD45+ cells. **(C)** Count of M1-TAMs per g tumor. **(D)** Count of M2-TAMs per g tumor. **(E)** Percentage of MDSCs among CD45+ cells in tumor tissue. **(F)** Count of MDSCs per g tumor. **(G)** Percentage of M-MDSCs among CD45+ cells in tumor tissue. **(H)** Count of M-MDSCs per g tumor. Data are presented as mean ± SEM (n = 4 per group). "ns" indicates no statistically significant difference, while asterisks indicate statistically significant difference (*, *P* < 0.05).
