# Supplementary material for: Plasmodium infection fully activates the immune system in peripheral blood and tumor microenvironment in a murine Lewis lung cancer model
Source: Front Mol Biosci. 2026 Jan 28;12:1724792. doi: 10.3389/fmolb.2025.1724792 (PMC12892102; doi:10.3389/fmolb.2025.1724792)
Supplement: Supplementary file 2 [file Supplementaryfile2.docx]

**Supplementary Table 1.** Flow Cytometry Antibodies

| Target | Fluorochrome | Catalog Number | Manufacturer | Volume (μL) |
| --- | --- | --- | --- | --- |
| CD45 | eFlour 506 | 69*-*0451*-*82 | eBioscience | 0.625 |
| CD3 | AF488 | 100210 | BioLegend | 0.250 |
| CD4 | APC/Fire810 | 100480 | BioLegend | 0.150 |
| CD8 | BV570 | 100740 | BioLegend | 0.625 |
| CD11b | APC/Fire750 | 101262 | BioLegend | 0.313 |
| CD44 | BV711 | 103057 | BioLegend | 0.156 |
| CD49d | PE*-*Cy7 | 103618 | BioLegend | 0.156 |
| CD62L | BV650 | 104453 | BioLegend | 1.250 |
| CD69 | AF647 | 104518 | BioLegend | 0.500 |
| CD86 | PE*-*Cy5 | 105016 | BioLegend | 1.250 |
| CD127 | BV421 | 135027 | BioLegend | 1.250 |
| CD206 | AF700 | 141734 | BioLegend | 0.250 |
| F4/80 | BV605 | 123133 | BioLegend | 1.250 |
| KLRG1 | PE | 138408 | BioLegend | 0.313 |
| Ly6C | PE/Dazzle 594 | 128044 | BioLegend | 0.300 |
| Ly6G | PE/Fire810 | 127673 | BioLegend | 1.250 |
| MHC II | eFlour 450 | 48*-*5321*-*82 | eBioscience | 0.625 |
| PD*-*1 | PerCP*-*eFlour 710 | 46*-*9985*-*82 | eBioscience | 0.625 |
| Fc Block | N/A | 156604 | BioLegend | 0.25 μg/test |

Note: All antibodies were used in a total staining volume of 100 μL per sample. The listed volume indicates the amount used per test. Fc Block was applied at 0.25 μg per test to prevent non*-*specific binding.

**Supplementary Table 2.** Definitions of Immune Cell Subset Populations

| **Population** | **Abbreviation** | **Characteristics** | **Function** | **Surface Marker Profile** |
| --- | --- | --- | --- | --- |
| Naive CD4+ T cell | CD4+ naive | Have not yet encountered an antigen and have high proliferative potential. | Differentiate into various types of effector or memory T cells upon antigen exposure. | CD45+CD3+CD4+CD44loCD62L+CD69- |
| Central memory CD4+ T cell | CD4+ Tcm | Strong self-renewal and proliferative capabilities and mainly reside in lymphoid tissues. | Rapidly differentiate into effector T cells upon re-exposure to the antigen. | CD45+CD3+CD4+CD44hiCD62L+CD69- |
| Effector memory CD4+ T cell | CD4+ Tem | Strong effector functions and mainly reside in peripheral tissues. | Rapidly respond to antigen stimulation and secrete cytokines. | CD45+CD3+CD4+CD44hiCD62L-CD69- |
| Tissue^-^resident memory CD4+ T cell | CD4+ Trm | Reside long-term in peripheral tissues (e.g., skin, mucosa) and do not enter the bloodstream. | Provide rapid local immune responses. | CD45+CD3+CD4+CD44hiCD62L-CD69+ |
| Naive CD8+ T cell | CD8+ naive | Have not yet encountered an antigen and have high proliferative potential. | Differentiate into various types of effector or memory T cells upon antigen exposure. | CD45+CD3+CD8+CD44loCD62L+CD127+KLRG1- |
| Central memory CD8+ T cell | CD8+ Tcm | Strong self-renewal and proliferative capabilities and mainly reside in lymphoid tissues. | Rapidly differentiate into effector T cells upon re-exposure to the antigen. | CD45+CD3+CD8+CD44hiCD62L+CD69-CD49dhi |
| Effector memory CD8+ T cell | CD8+ Tem | Strong effector functions and mainly reside in peripheral tissues. | Rapidly respond to antigen stimulation, secrete cytokines, and kill target cells. | CD45+CD3+CD8+CD44hiCD62L-CD69-CD49dhi |
| Tissue^-^resident memory CD8+ T cell | CD8+ Trm | Reside long-term in peripheral tissues (e.g., skin, mucosa) and do not enter the bloodstream. | Provide rapid local immune responses. | CD45+CD3+CD8+CD44hiCD62L-CD69+CD49dhi KLRG1- |
| Virtual memory CD8+ T cell | CD8+ Tvm | Reside long-term in the endothelium of blood vessels and have rapid response capabilities. | Rapid immune surveillance and responses within the vasculature. | CD45+CD3+CD8+CD44hiCD62L+CD69+CD49dlo |
| Short-lived effector CD8+ T cell | CD8+ SLEC | High cytotoxicity but a short lifespan. | Rapidly clear pathogens during acute infections. | CD45+CD3+CD8+CD44hiCD62L+CD127-KLRG1+CD49dhi |
| Memory precursor CD8+ T cell | CD8+ MPEC | Have effector functions but also retain some memory cell characteristics, allowing them to survive long-term. | Play a crucial role in chronic infections, providing sustained effector cell production. | CD45+CD3+CD8+CD44hiCD62L+CD127+KLRG1-CD49dhi |
| M1 macrophage | M1 | Classically activated macrophages that play a crucial role in the immune response against pathogens and in inflammation. | Produce pro-inflammatory cytokines such as TNF-α, IL-6, and IL-12, and express high levels of MHC II molecules, making them efficient antigen-presenting cells. | CD45+CD3-CD11b+F4/80+CD86+CD206- |
| M2 macrophage | M2 | Alternatively activated macrophages involved in tissue repair, wound healing, and immune regulation. | Secrete anti-inflammatory cytokines such as IL-10 and TGF-β, and they express markers like CD206 (mannose receptor) and CD163. | CD45+CD3-CD11b+F4/80+CD206+ |
| Monocytic myeloid-derived suppressor cell | M*-*MDSC | Suppress immune responses and are often found in the context of cancer and chronic infections. | Inhibit T cell activation and proliferation through the production of immunosuppressive molecules such as arginase, iNOS, and TGF-β. | CD45+CD3^-^CD11b+MHC II-Ly6G-Ly6Chi |
| Polymorphonuclear myeloid^-^derived suppressor cell | PMN*-*MDSC | Have immunosuppressive functions. They are characterized by a neutrophil-like morphology. | Suppress T cell responses, particularly by inhibiting the production of TNF-α and IFN-γ. They can also induce T cell apoptosis. | CD45+CD3-CD11b+MHC II- Ly6G+Ly6Clo |

Note: hi and lo indicate high and low expression levels of marker molecules, respectively. + and - indicate expression and no expression of the marker molecules, respectively. All populations were pre*-*gated on live, single cells.

**Supplementary Table 3.** Alterations of CD4+ and CD8+ T Cell Subsets

| **Subsets** | **Percentage** | | **Count** | | **PD-1** | |
| --- | --- | --- | --- | --- | --- | --- |
|  | **Blood** | **Tumor** | **Blood** | **Tumor** | **Blood** | **Tumor** |
| CD4+ T | ⬇ | ns | ⬆ | ⬆ | ⬆ | ⬇ |
| CD4+ naive | ⬇ | ns | ⬇ | ns | ⬆ | ns |
| CD4+ Tcm | ⬆ | ns | ⬆ | ⬆ | ⬆ | ns |
| CD4+ Tem | ⬆ | ns | ⬆ | ⬆ | ⬆ | ns |
| CD4+ Trm | nd | ⬇ | nd | ⬆ | nd | ⬇ |
| CD8+ T | ⬆ | ⬆ | ⬆ | ⬆ | ⬆ | ⬇ |
| CD8+ naive | ⬇ | ⬇ | ⬆ | ⬆ | ns | ns |
| CD8+ Tcm | ⬆ | ns | ⬆ | ⬆ | ns | ⬆ |
| CD8+ Tem | ⬆ | ⬆ | ⬆ | ⬆ | ns | ⬇ |
| CD8+ Trm | nd | ⬇ | nd | ⬆ | nd | ⬇ |
| CD8+ Tvm | ⬆ | ⬇ | ⬆ | ns | ⬆ | ns |
| CD8+ SLEC | ⬆ | ⬆ | ⬆ | ⬆ | ⬆ | ns |
| CD8+ MPEC | ⬆ | ⬇ | ⬆ | ns | ⬆ | ns |

Note: naïve: naïve T cell; Tcm: central memory T cell; Tem: effector memory T cell; Tvm: virtual memory T cell; Trm: tissue resident memory T cell; SLEC: short-lived effector T cell; MPEC: memory precursor T cell. ⬆: increase; ⬇: decrease; ns: no significant difference between groups; nd: no data. Percentage: the percent of CD4+ T subset in CD4+ T, or the percent of CD8+ T subset in CD8+ T. Count: the absolute number of T cell subset per μL peripheral blood or the absolute number of T cell subset per g tumor.
